# AncestryGeni: a novel genetic ancestry classification pipeline for small and noisy sequence data

**DOI:** 10.1093/bioinformatics/btaf391

**Published:** 2025-07-08

**Authors:** Eran Elhaik, Sara Behnamian, Michael Howe, Hongwei Tang, Huihuang Yan, Shulan Tian, Suganti Shivaram, Cinthya Zepeda Mendoza, Kylee MacLachlan, Saad Usmani, Mehdi Pirooznia, Gareth Morgan, Patrick Blaney, Francesco Maura, Linda B Baughn

**Affiliations:** Department of Biology, Lund University, Lund 22362, Sweden; Centre for GeoGenetics, Globe Institute, University of Copenhagen, Copenhagen, 1350, Denmark; Pioneer Centre for AI, University of Copenhagen, Copenhagen, 1350, Denmark; Division of Hematology, Department of Internal Medicine, Mayo Clinic, Rochester, MN, 55905, United States; Division of Hematopathology, Department of Laboratory Medicine and Pathology, Mayo Clinic, Rochester, MN, 55905, United States; Division of Computational Biology, Department of Quantitative Health Sciences, Mayo Clinic, Rochester, MN, 55905, United States; Division of Computational Biology, Department of Quantitative Health Sciences, Mayo Clinic, Rochester, MN, 55905, United States; Division of Hematopathology, Department of Laboratory Medicine and Pathology, Mayo Clinic, Rochester, MN, 55905, United States; Division of Hematopathology, Department of Laboratory Medicine and Pathology, Mayo Clinic, Rochester, MN, 55905, United States; Myeloma Service, Department of Medicine, Memorial Sloan Kettering Cancer Center, New York, NY, 10065, United States; Myeloma Service, Department of Medicine, Memorial Sloan Kettering Cancer Center, New York, NY, 10065, United States; School of Medicine, Johns Hopkins University, Baltimore, MD, 21205, United States; Interventional Oncology, Johnson & Johnson Enterprise R&D, Raritan, NJ, 08869, United States; Multiple Myeloma Research Program, Perlmutter Cancer Center, NYU Langone Medical Center, New York, NY, 10016, United States; Multiple Myeloma Research Program, Perlmutter Cancer Center, NYU Langone Medical Center, New York, NY, 10016, United States; Myeloma Service, Department of Medicine, Memorial Sloan Kettering Cancer Center, New York, NY, 10065, United States; Division of Hematopathology, Department of Laboratory Medicine and Pathology, Mayo Clinic, Rochester, MN, 55905, United States

## Abstract

**Motivation:**

Efforts to address health disparities are often limited by the lack of robust computational tools for inferring genetic ancestry by calculating an individual’s genetic similarity to continental groups. We have already shown that a preferred alternative to self-described race is using ancestry-informative markers (AIMs) that can be classified into ancestral components and used to estimate their similarity to those of known populations to identify continental groups. However, real-world genomic data can present challenges, including limited availability of germline DNA, a small number of AIMs for each sample, and the use of different variant calling software, limiting the application of existing solutions.

**Results:**

Here, we describe a novel supervised machine-learning tool *AncestryGeni*, which infers genetic ancestry for samples with even a hundred markers and is applicable to any genomic data, including whole exome sequencing (WES) and RNA sequencing (RNA-Seq) data. Applying *AncestryGeni* to a real-world genomic dataset obtained from the Multiple Myeloma Research Foundation (MMRF) CoMMpass study, we show that it is more accurate than the commonly used FastNGSadmix when using nonstandard genomic material. We also demonstrate that when using *AncestryGeni*, the tumor-derived sequence obtained from WES and RNA-Seq can be a robust data source to accurately estimate an individual’s genetic similarity to a continental group.

**Availability and implementation:**

AncestryGeni pipeline is available at https://github.com/eelhaik/AncestryGeni/tree/main.

## 1 Introduction

Genomic differences observed between individuals have been documented to be relatively small, with the greatest variations typically observed when comparing individuals from different continents (Elhaik 2012). These variants can impact many aspects of human health, from differences in the distribution of causal variants associated with a disease phenotype to variations in drug response (Yusuf and Wittes 2016, Sirugo *et al.* 2019). However, not all continental groups have been studied equally, and their unique genetic risks have not been sufficiently assessed, limiting our ability to comprehensively understand health disparities (Popejoy and Fullerton 2016).

Health disparity research has historically relied on the use of self-reported race data. Self-reported race refers to how individuals classify themselves based on socially and culturally constructed categories. This approach may be inaccurate as people may confuse ancestral perceptions with true heritage, making genetic inferences of ancestry preferable and unbiased. Genetic ancestry refers to the combination of individual gene pools that reflect the individual’s DNA lineage, including the subset of paths by which DNA in each genome has been inherited autosomally. Genetic ancestry can be summarized into groups or categories of interest, defined in the context of specific timeframes and geographical locations, including continents (Elhaik *et al.* 2014). When an individual shares genetic similarity to a present-day reference population from a continental group, they may exhibit high genetic similarity to that continental group (Mathieson and Scally 2020, Coop 2022, National Academies of Sciences, Engineering, and Medicine 2023). While this approach is preferable, efforts to address health disparities in medical research are often limited by the lack of robust computational tools to analyze individuals in heterogeneous datasets. Moreover, germline whole genome sequencing (WGS) or microarray data suitable for inferring genetic ancestry are often unavailable. Oftentimes, tumor-derived sequencing data from whole exome sequencing (WES) or RNA sequencing (RNA-Seq) are the only available genomic sources, representing unique challenges unaddressed by existing genetic ancestry tools.

Admixture or admixture-like analyses originated in the popular program STRUCTURE (Falush *et al.* 2003). Here, the ancestry of each individual is modeled as a proportion of *K* admixture components, which allegedly represent “historical populations” in the model. This form of global ancestry is implemented by tools like *FRAPPE* or *ADMIXTURE*, which are limited in their ability to analyse extremely small and noisy datasets and convert their admixture results into continental group data (Tang *et al.* 2005). This limitation is more pronounced with local ancestry tools like ChromoPainter (Lawson *et al.* 2012) or LAMP (Baran *et al.* 2012), which require dense genome-wide data to identify haplotypes from various ancestries (Carress *et al.* 2021). To the best of our knowledge, no tool exists that can detect regional gene pools and convert them into continental groups, applicable to any genomic data and operational on noisy data that contain a very small number of single nucleotide polymorphisms (SNPs).

Our previous solutions to calculating genetic ancestry have relied on ∼130 000 ancestry-informative markers (AIMs) from which nine gene pools were calculated. Genetic ancestry could be obtained from these gene pools, even if as little as 1000 AIMs were available, and the samples could be geographically localized with high accuracy (Elhaik *et al.* 2014, Elhaik and Ryan 2019). However, the challenges presented with real-world genomic data, namely the variability in number of markers available for each sample, the small number of markers, genotype differences due to the use of different variant calling software, and genotype differences in the somatic and germline samples, limit the applicability of existing solutions to calculating genetic ancestry as done in previous studies (Baughn *et al.* 2018, 2020) and require new tools that rely on a large number of SNPs designed for redundancy.

**Figure 1.**
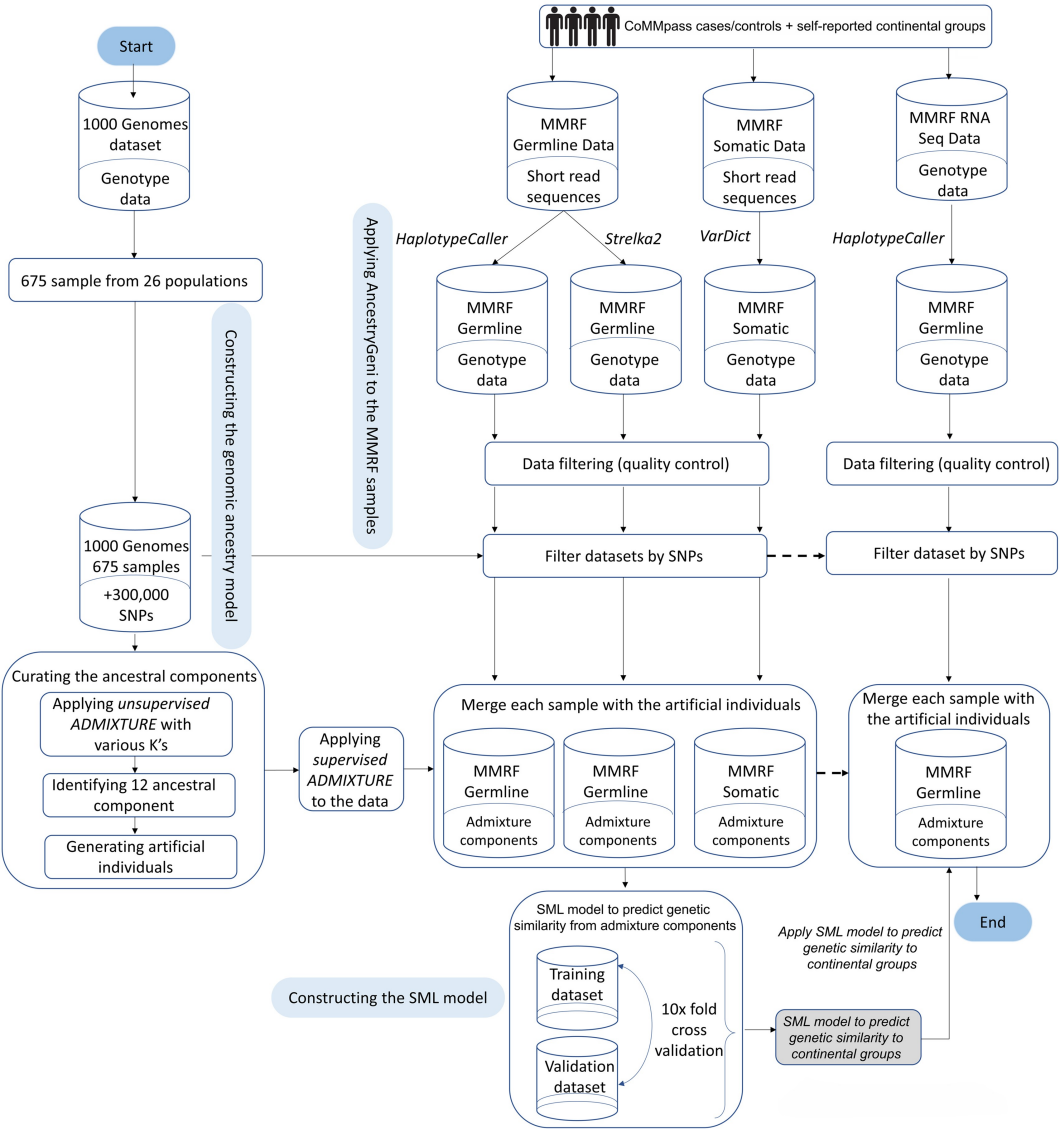
*AncestryGeni* workflow. Schematic overview of the detection of an individual’s genetic similarity to a continental group workflow with *AncestryGeni* and how to apply it to identify the continental group genetic similarity from both WES and RNA-Seq data.

Here, we describe the development of a novel algorithm *AncestryGeni* (Fig. 1), which calculates genetic ancestry using 12 gene pools and classifies the continental group of individuals. We applied *AncestryGeni* to a real-world genomic dataset (WES and RNA-Seq) of tumor-derived sequence data obtained from the Multiple Myeloma Research Foundation (MMRF) CoMMpass study (Skerget *et al.* 2024). This unique dataset was selected because multiple myeloma (MM) is a prevalent hematologic malignancy affecting continental groups disproportionally (Siegel *et al.* 2024). African American (AA) individuals have the highest incidence of MM and its precursor condition, monoclonal gammopathy of undetermined significance (MGUS), of any self-report race group in the US (Siegel *et al.* 2024) with a ∼1.5–2-fold higher age-adjusted incidence compared to European American (EA) individuals (Weiss *et al.* 2009, Landgren *et al.* 2009a, 2009b, Greenberg *et al.* 2012). A ∼2-fold increased prevalence of MGUS has also been identified in Africans from Ghana compared to EA individuals from Minnesota (Landgren *et al.* 2007). In addition, AA populations are likely to be admixed with ancestry from more than one genetic ancestry group (Bryc *et al.* 2015), and the dataset included multiple tumor-derived sequence data types along with germline sequence and self-report continental group data. Because the increased risk for MGUS was identified in AA and African individuals, we further summarized genetic ancestry into the continental groups. Using *AncestryGeni*, we demonstrate that the tumor-derived sequence data obtained from WES and RNA-Seq can be a robust genetic source to calculate genetic similarity, which can be used to estimate an individual’s genetic ancestry and assign the individual into a continental group.

## 2 Materials and methods

### 2.1 Constructing the genomic components

To create genomic (ancestral) components, we randomly selected over 300 000 SNPs from the 1000 Genomes Project that were sequenced in all 26 reference populations, of which we selected 25 random samples of each population and an overall 675 samples. We then used unsupervised ADMIXTURE (v1.3.0) (Alexander and Lange 2011) to analyse 15 values of *K* (5–20), identifying *K *= 12 as the optimal choice for producing components with the best geographical clustering and minimal noise. This was done by observing the proportion of the components in the 1000 Genomes populations and selecting a *K* that produced geographically homogeneous and genetically meaningful components. Applying a 0.1 threshold to each ancestral component at the end of the pipeline removed minor contributions. Using ADMIXTURE’s allele frequencies output (*p*-file), 15 synthetic samples associated with each genomic component were generated for each component (Elhaik *et al.* 2014, Esposito *et al.* 2018, Behnamian *et al.* 2022). Supervised ADMIXTURE was then applied to any sample of matching SNPs with respect to the synthetic samples, as in Elhaik and Ryan (2019), to calculate the genomic components.

### 2.2 Adapting a SML model to classify the continental groups from genomic components

To test which supervised machine learning (SML) model can best classify individuals into the continental groups from the genomic components, we evaluated the most common SML algorithms. For each model, we assessed key performance metrics such as accuracy, AUC, recall, precision, *F*1 score, and computational efficiency when the training set was 1K SNP dataset, and the test set was 300K SNP dataset. The comparison results for other training and testing sets were similar and are not shown. The LDA algorithm outperformed all the other SMLs.

LDA’s foundation traces back to Fisher’s 1936 seminal work, where he introduced linear discriminant analysis (LDA) to classify species based on their morphological traits. Its ability to maximize between-class variance while minimizing within-class variance ensures optimal separation of classes, making it an effective choice for tasks involving high-dimensional genetic data (Fischer 1936). LDA performs classification by constructing optimal decision boundaries that maximize separation between classes while minimizing variation within each class. As input features, we used genomic components collapsed into five continental groups: Africa, East Asia, Europe, Central Asia, and America. The algorithm projects these high-dimensional genomic features onto a lower-dimensional space where class differences are most pronounced. Although LDA assumes balanced class distributions and equal variance among classes, and our genomic dataset had uneven sample sizes across the continental groups, LDA proved effective at identifying distinctive patterns in the ancestry data, likely due to its strong statistical foundation in covariance analysis and dimensionality reduction. While the uneven class distribution may impact classification accuracy for the smaller continental groups, we demonstrated that LDA is suitable for genetic ancestry classification even in small sample sizes.

Prior to applying LDA, each dataset was randomly split, with 50% of the samples allocated for training and validation and the remaining 50% used for testing. A default threshold of 0.1 was applied to all genomic components, followed by normalization. We found that after filtering by read quality, this is the optimal threshold for the kind of data in CoMMpass. However, if it resulted in null (i.e., if all the genomic components are below 0.1), a threshold was lowered by 0.01, and the process was repeated until a threshold could be applied to the entire data, followed by normalization. Within the training set, 10-fold cross-validation was employed to evaluate the performance of LDA model. The genomic data were divided into 10 subsets; nine subsets were used for training, and the remaining subset was used for validation. This process was repeated 10 times, ensuring that each subset served as the validation set once.

LDA hyperparameter optimization was conducted through an extensive grid search evaluating singular value decomposition (SVD), Least Squares (LSQR), and Eigenvalue solvers, along with varying shrinkage configurations (automatic and fixed values between 0 and 1), covariance storage options (True/False), and tolerance thresholds (ranging from 10^−4^ to 10^−1^). Performance evaluation utilized multiple metrics, including balanced accuracy, precision, recall, *F*1 score, and Matthews correlation coefficient (MCC). The optimal configuration, utilizing the LSQR solver with automatic shrinkage, enabled covariance storage and tolerance of 0.0001, achieving 72.76% balanced accuracy on the test set while maintaining consistent performance between the training and testing phases. This configuration demonstrated consistent performance between the training and testing on the 1000 Genomes Project dataset, utilizing 1K SNPs for training and 300K SNPs for testing, highlighting its robustness despite the disparity in SNP set sizes. Default parameters demonstrated comparable or superior performance in specific scenarios, suggesting LDA’s inherent robustness across different genomic data patterns. The systematic grid search methodology ensured that the selected parameters were effectively generalized across diverse SNP datasets and class distributions, balancing model complexity with classification performance.

We implemented an LDA classifier with normalized genomic components. The model utilizes the 12 components (Africa A, Africa B, Central Asia, Central Europe, East Asia, Far East Asia, India, Native America, Scandinavia, Southeast Asia, South Europe, and Western Europe) as features. We applied a normalization procedure with a threshold parameter *τ* = 0.1 to ensure all components sum to one for each individual. For model validation, we employed a stratified *k*-fold cross-validation (*k *= 5) with a 0.2 test fraction. LDA’s performance was evaluated using standard classification metrics, including accuracy, area under the receiver operating characteristic curve (AUROC), precision, recall, *F*1-score, Cohen’s Kappa coefficient, and MCC to classify individuals’ ancestry into five major continental groups: African, American, Central Asian, Eastern Asian, and European populations. The LDA classifier learned to construct decision boundaries that maximize class separability during each iteration. After all folds were completed, the model’s overall performance was evaluated by aggregating metrics such as accuracy, precision, and recall across all iterations. This approach allowed the model to generalize effectively and reduced the risk of overfitting, even in the presence of uneven class distributions in the dataset. When provided with genomic components of the test sample, *AncestryGeni* identifies the continental groups.

### 2.3 Evaluating the accuracy of *AncestryGeni* on 1000 Genomes Data

We down-sampled the 1000 Genomes dataset calculated for over 300K SNPs by randomly selecting 50K, 10K, 1K, and 100 random SNPs and recalculating the genetic ancestry of the samples. We then applied the process above to these cohorts, using 50% of one cohort for training and the remaining cohorts for testing, and calculated the confusion matrices. Classification accuracy was calculated by dividing the total number of correct classifications by the total number of classifications made by the model. To estimate the significance, we tested the accuracy of the classified data against the null hypothesis (50%) using a chi-squared test. To further evaluate *AncestryGeni’s* performance across different SNP densities, we generated ROC curves (Green and Swets 1966), in a multi-class setting using a one-vs-rest (OvR) strategy, where each ancestry group was evaluated against all others independently, and precision–recall curves (Davis and Goadrich 2006) using the same dataset configurations described above. These complementary metrics evaluate the model’s classification abilities across varying genetic marker densities and ancestral populations.

### 2.4 Evaluating the accuracy of *AncestryGeni* on perturbed data

To assess the robustness of *AncestryGeni* to perturbed data, we added between 1% and 10% SNPs (based on the number of SNPs in a sample) as noise to RNA-Seq samples. After excluding Amerindian samples from the test set, as these were not part of the model’s training parameters in the case of RNA-Seq samples, 271 balanced samples were retained per perturbation level. For each sample, those SNPs are called from other samples but not from themselves. To generate a set of VCF files that carry variable perturbation levels for each of the autosomes, we first compiled a list of unique SNPs by combining SNPs from all samples. If a SNP was called from multiple samples, the one with the highest quality was retained. For each chromosome, we randomly sampled 1–10% SNPs (with 1% step size) from the above list of SNPs after excluding those present in that sample. At each perturbation level (1–10%). We added the randomly sampled SNPs from all 22 chromosomes to the original VCF, resulting in 10 modified VCF files per sample. *AncestryGeni* was executed on the modified and original VCF files.

For performance quantification, we implemented a multi-metric evaluation framework. The classifier’s discriminative power was assessed through ROC-AUC scores using OvR multi-class strategy, where the model computed probabilities for each ancestry component against all others collectively. Complementary metrics included weighted precision and recall (measuring classification exactness and completeness), *F*1 scores (harmonic mean of precision–recall), Matthews correlation coefficient (for balanced performance assessment), and Cohen’s Kappa (for reliability beyond chance). Performance trajectories were systematically tracked across the perturbation spectrum (f0–f9), enabling detailed analysis of classification robustness under progressive signal perturbation.

### 2.5 Read quality assessment, mapping, and variant calling workflow

FastQC (v0.11.8, www.bioinformatics.babraham.ac.uk/projects/fastqc) was utilized to assess the base quality of reads. Trimmomatic (v0.39) (Bolger *et al.* 2014) was used to remove adapters, followed by reads mapping to the GRCh38 genome reference with Burrows–Wheeler Aligner (BWA-MEM, v0.7.17) (Landgren *et al.* 2009b, Li and Durbin 2010). Picard (v2.27.5, https://github.com/broadinstitute/picard) was used to remove PCR duplicates. Reads with mapping quality below 30 were filtered out, and the Genome Analysis Toolkit (GATK, v4.2.4.1) was used for base quality recalibration. Germline variants were called from non-tumor samples in the CoMMpass cohort using Strelka2 (v2.9.9) (Kim *et al.* 2018) and HaplotypeCaller (v4.3.0.0) (Poplin *et al.* 2018), and all variants called from the tumor samples using VarDict (v1.8.3) (Lai *et al.* 2016). For the RNA-Seq data, we called variants using HaplotypeCaller. Variants were annotated using the web server of variant effect predictor (release 111, https://useast.ensembl.org/Tools/VEP) (Hunt *et al.* 2022). To minimize false positive calls, only variants detected at sites with total reads ≥6 and supporting reads ≥2 for the variant allele were retained.

### 2.6 Identifying MMRF CoMMpass samples

A total of 448 tumor RNA-Seq, germline or tumor WES, or germline WGS samples obtained from patients with MM through the CoMMpass study (NCT01454297) were selected for evaluation by *AncestryGeni*. Self-identified continental groups included 348 European, 90 African (AA), and 10 Asian. The CoMMpass study was approved by ethics committees or institutional review boards (IRB) at individual study sites.

### 2.7 Calculating genetic ancestry for the CoMMpass samples

To calculate the genetic ancestry, we selected all bi-allelic SNPs that passed the filters (=PASS) and had a quality score (=QUAL) of 40 or higher for further processing. For each sample, we identified all SNPs that overlapped with the SNP set of the synthetic samples and created a merged file to which we applied Supervised ADMIXTURE to calculate the admixture proportion for that sample. The same step was applied to RNA-Seq data. We then applied the process above to these cohorts, using 50% of one cohort for training and the remaining cohorts for testing, and calculated the confusion matrices.

### 2.8 MGP1000 genetic ancestry analysis

MGP1000 includes a genetic ancestry estimation workflow within the germline module to determine the proportion of genetic similarity per patient from distinct reference populations. The workflow begins with using ANGSD v0.941 (Korneliussen *et al.* 2014) to calculate genotype likelihoods at a set of high-quality genome-wide SNPs well represented across each reference population. The reference population from the 1000 Genomes Project (Genomes Project *et al.* 2015) includes five continentally distinct super-populations, each comprising 23 unique sub-continental populations. Lastly, the tool FastNGSadmix v1.0 (Jorsboe *et al.* 2017) (http://www.popgen.dk/software/index.php/FastNGSadmix) uses the genotype likelihoods at each SNP and allele frequencies of reference population in an expectation–maximization algorithm to determine admixture proportions.

## 3 Results

### 3.1 Continental group classification using FastNGSadmix in comparison to self-reported data

**Figure 2.**
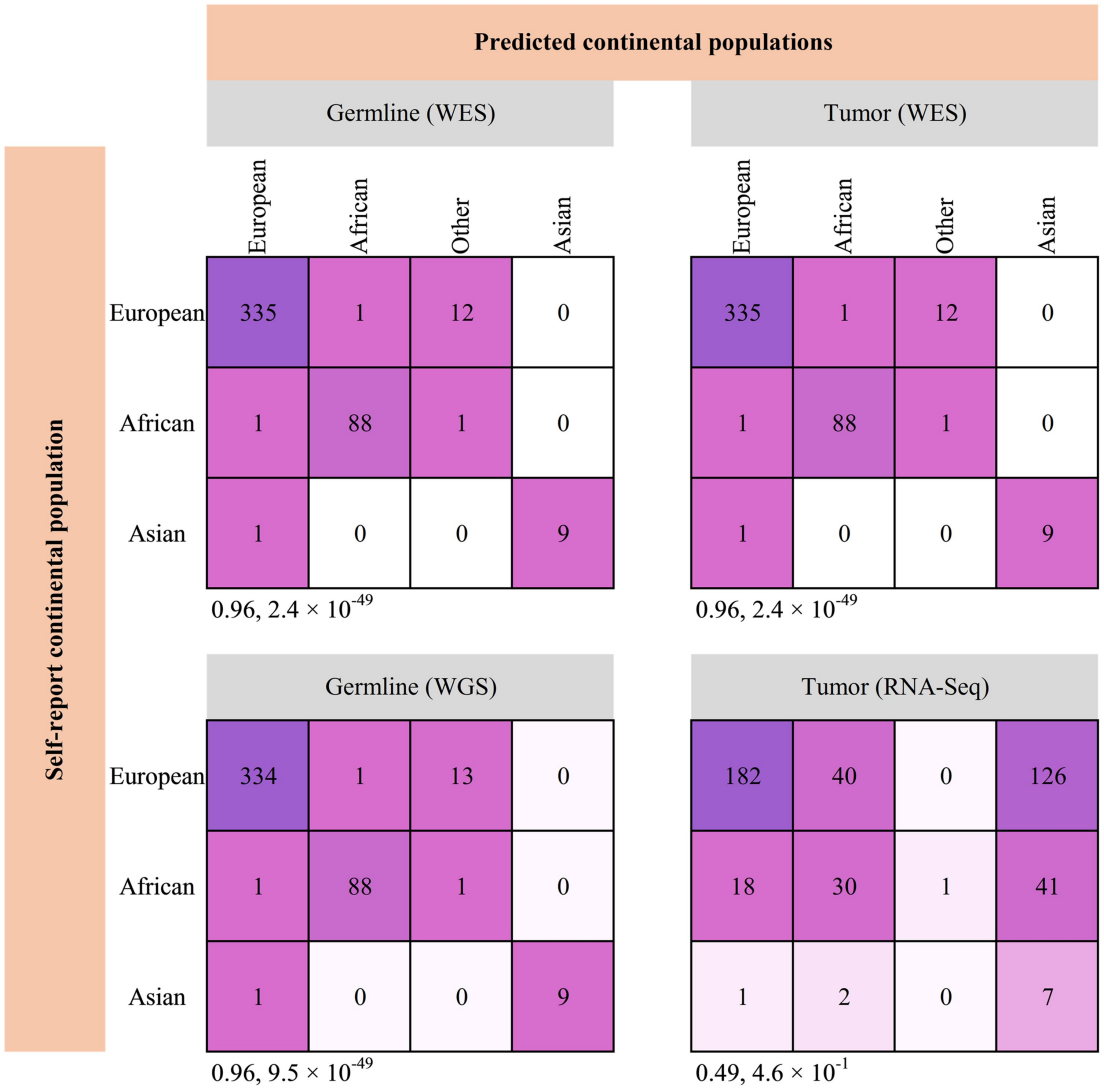
Confusion matrices to evaluate the accuracy of FastNGSadmix in detecting an individual’s genetic similarity to continental groups from the CoMMpass cohort. FastNGSadmix was applied to 448 germline WES, germline WGS, tumor WES, and tumor RNA-Seq, and the calculated genetic similarity to continental groups (top header) was compared to the self-reported continental groups (side header). Accuracy and *P*-value are shown under each confusion matrix.

To test which genomic data can be used as input for estimating an individual’s genetic similarity to a continental group, we first evaluated the performance of a commonly used algorithm, FastNGSadmix, in classifying individuals to the continental groups when multiple data modalities are used. We applied FastNGSadmix to WGS, WES, and RNA-Seq data from germline or tumor sources from the CoMMpass study and compared the continental group with self-reported data. FastNGSadmix achieved high accuracy (0.96) when applied to WGS and WES data, regardless of whether the DNA was obtained from germline or tumor (Fig. 2), but low accuracy (0.49) when applied to RNA-Seq data. We reasoned that the poor performance of FastNGSadmix is likely due to its training on germline reference populations, which contain more ancestral identifiable information than found in short coding regions. Here, we sought to develop a method that could be trained on any data modality, including RNA-Seq.

### 3.2 Development of *AncestryGeni*

We present *AncestryGeni*, a novel algorithm that calculates genetic ancestry and accurately identifies an individual’s genetic similarity to a continental group (Fig. 1). The rationale for developing *AncestryGeni* is supported by the observation that most human variation is within continental groups (Elhaik 2012) and is subjected to selection and genetic drift processes that modulate the allele frequencies over time (Graur *et al.* 2013). *AncestryGeni* leverages random markers and classifies them into predetermined gene pools, producing fingerprints unique to the continental groups. While the actual genetic ancestry proportions may exhibit high variation, particularly when there are only a small number of markers, most of this variation would be among the continental groups (Elhaik 2012), making it possible to train an SML algorithm on labeled data to recover the genetic similarity of an individual to a continental group.

**Figure 3.**
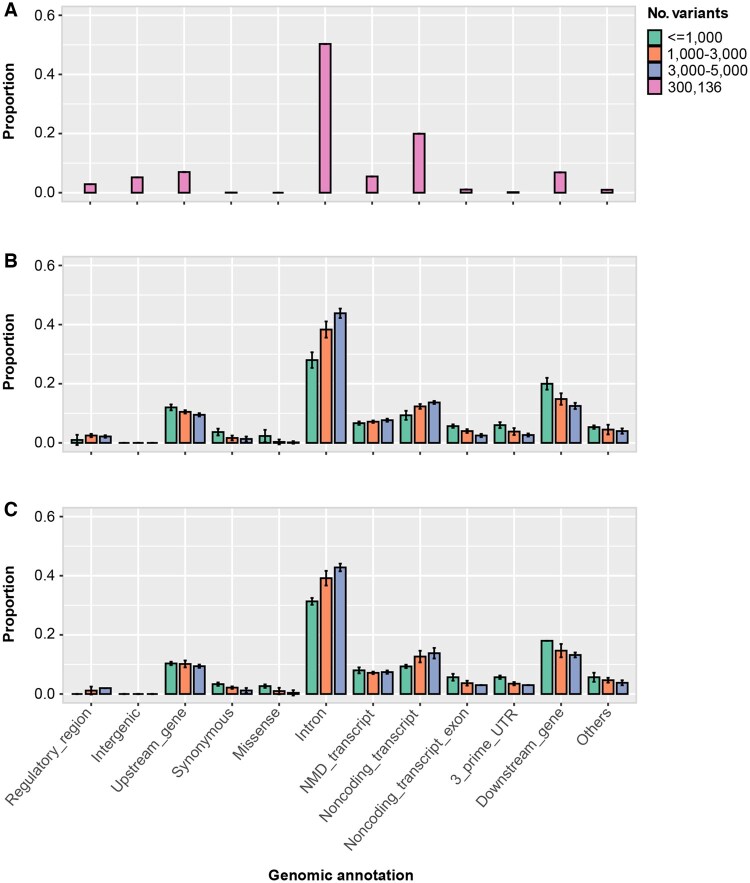
Annotation of variants called from RNA-Seq of CoMMpass samples. (A) The ∼300 000 reference SNPs from the 1000 Genomes Project. (B) Variants called from RNA-Seq samples with continental group similarity matching that from WES data. (C) Variants called from RNA-Seq samples with continental group similarity different from that based on WES data. Of the RNA-Seq samples, 99.4% had <5000 variants called by Strelka2. Samples were split into five subsets with ≤1000, 1000–2000, 2000–3000, 3000–4000, and 4000–5000 variants. Three samples (all samples if <3) were randomly selected from each subset, separately for samples with concordant continental group similarity and those with discordant continental group similarity. *Y*-axis represents mean ± sd in (B) and (C). NMD, nonsense-mediated mRNA decay.

**Figure 4.**
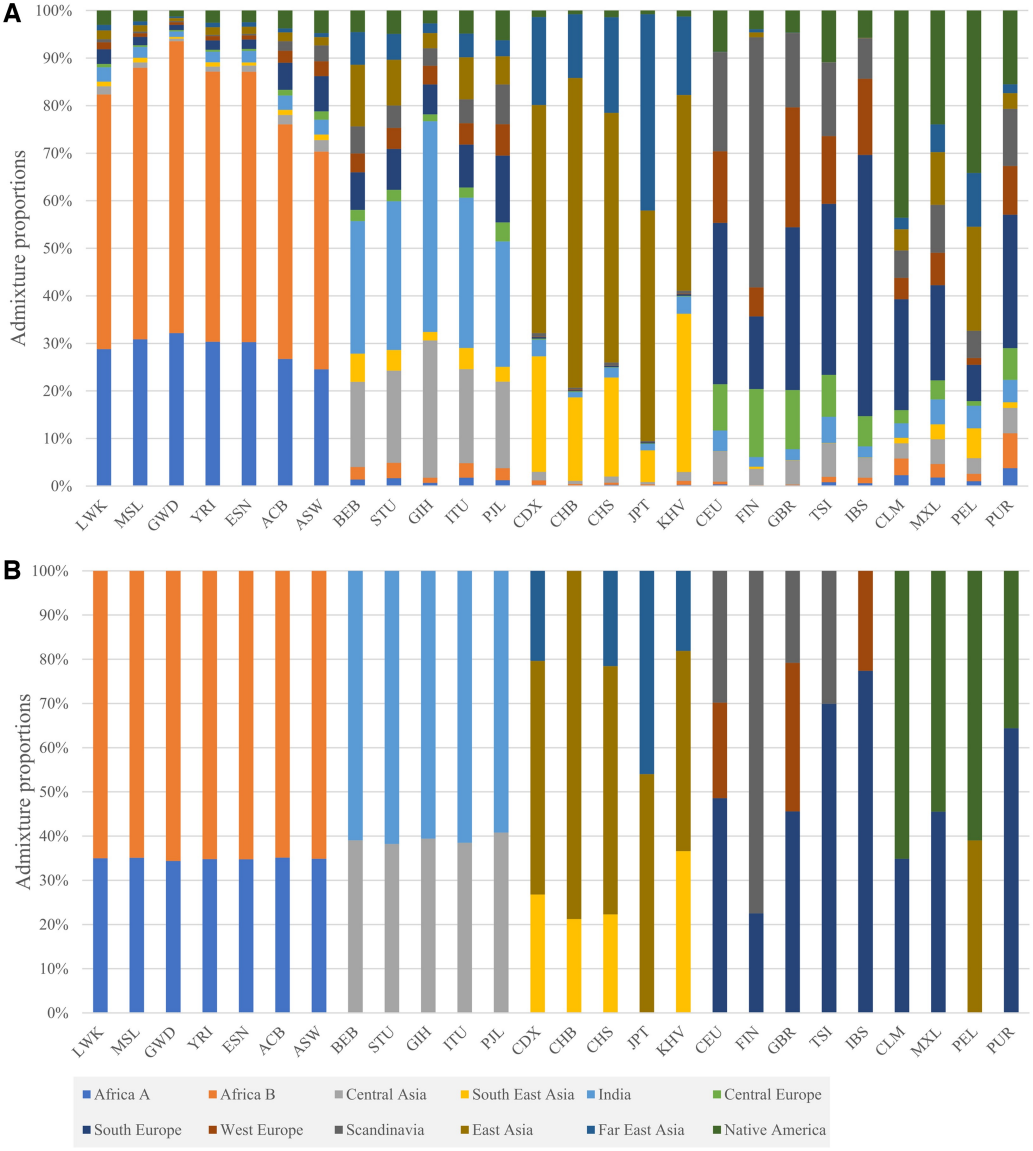
Twelve genetic (ancestry) components of the 1000 Genomes samples. The mean genetic components of 675 samples from the 1000 Genomes Project were calculated and plotted per population before (A) and after a 10% threshold was applied (B). Each vertical stacked bar represents a population, with colors corresponding to the 12 components. The plot demonstrates that the genomic components are geographically localized and can be used to identify continental groups.

We created a training dataset by randomly selecting over 300 000 SNPs from 675 (27%) out of 2504 samples representing all 26 reference populations from the 1000 Genomes Project (Sudmant *et al.* 2015). The number of SNPs was balanced between selecting a large number of markers while maintaining the performance of supervised ADMIXTURE (Alexander and Lange 2011), which calculates the admixture proportions of populations in relation to putative ancestral populations and includes over 7000 of the AIMs identified by Elhaik *et al.* (2014). As expected, most of the SNPs were intronic and noncoding transcripts, with a small proportion (0.15) within genes (Fig. 3). Next, we identified 12 gene pools representing different continents, including Africa, Europe, Asia, and the Americas. We generated diverse “in silico individuals” for each gene pool that allowed inferring the genetic similarity of test samples against the same 12 gene pools (Fig. 4). The gene pools created distinct signatures for each reference population (Fig. 4A) that allowed for the calculation of genetic similarity and classification into a continental group. To reduce the random noise due to the inclusion of ancestry uninformative markers, a threshold of 0.1 was applied for the genetic ancestry estimation of each sample (Fig. 4B).

### 3.3 *AncestryGeni* accurately classifies continental groups using SNP sets ranging from 100 to over 300 000

**Figure 5.**
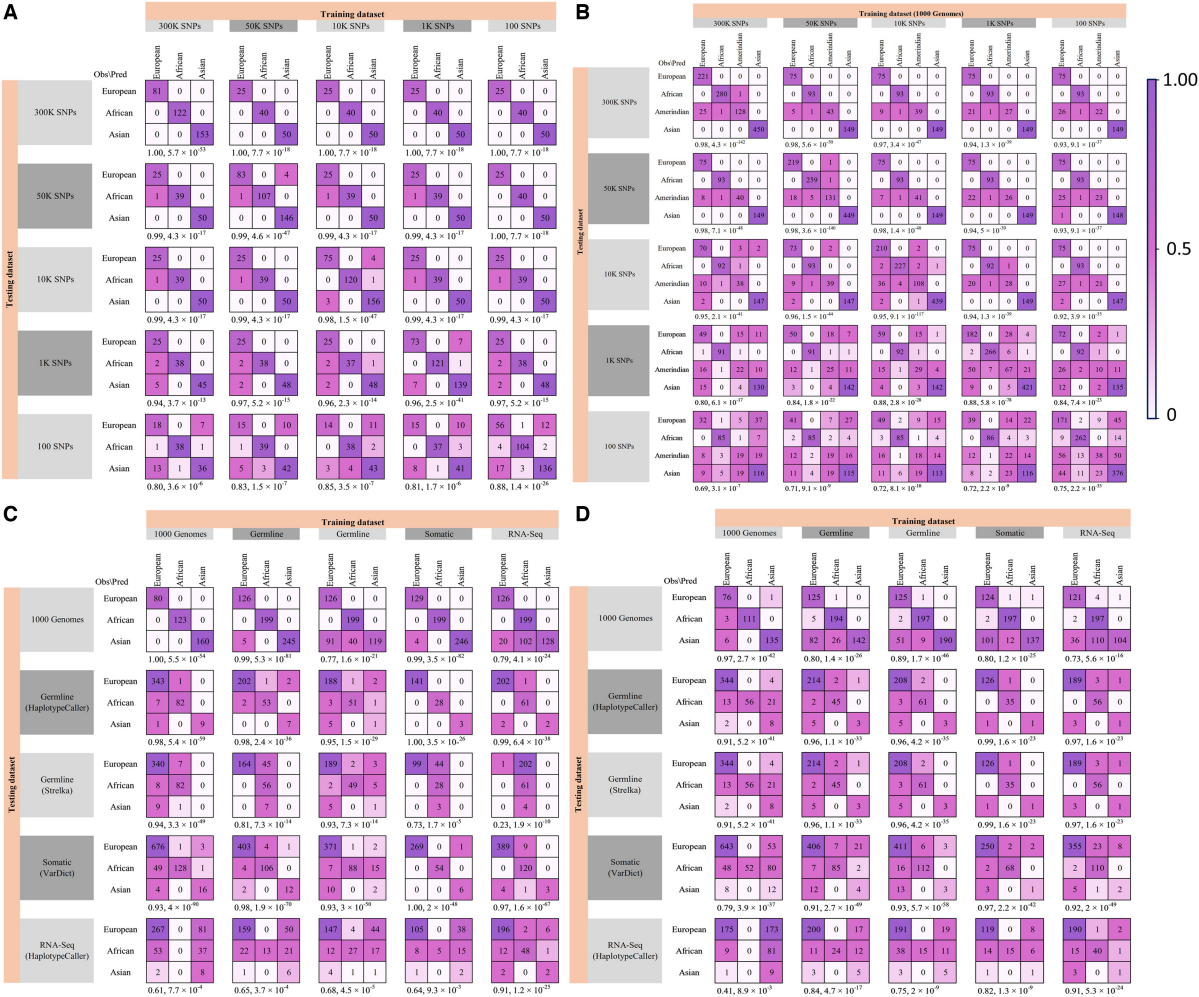
Confusion matrices to evaluate the classification accuracy of *AncestryGeni* applied to the 1000 Genomes and CoMMpass data. *AncestryGeni* was trained on 50% of the samples of one dataset (top header) and tested on non-overlapping samples of other datasets (rows). Amerindians were excluded from calculations (A), (C, D) due to their absence from the CoMMpass dataset, but they appear in (B). We first tested the classification accuracy of AncestryGeni for the 1000 Genomes dataset consisting of various number of SNPs. In (A), we trained it on 50% of the 675 samples used to calibrate the model dataset and tested it on the remaining 50% of the samples, and in (B), we trained it on 50% of 1829 non-overlapping samples that were not used for the gene pool design and tested on the remaining 50% of the samples. Next, we tested the model on the 1000 Genomes and CoMMPass datasets. In (C), we used the full SNP set for training and testing, and in (D), we used randomly selected 1200 SNPs (except the RNA-Seq data) for training and testing. Color gradients correspond to the percent of samples (scale is on the right). Accuracy and *P*-value are shown under each confusion matrix.

Next, we tested whether AncestryGeni could infer the continental group with high accuracy across SNP sets of variable sizes. While it is impossible to show that *AncestryGeni* is robust to any choice of SNPs, we can evaluate whether it yields accurate results for small random SNP sets. We down-sampled the 1000 Genomes training dataset by randomly selecting 50K, 10K, 1K, and 100 SNPs, calculated the genetic ancestry, and tested the accuracy of continental classification, focusing on the three continental groups (African, European, and Asian) represented in the CoMMpass test dataset. The genomic components calculated for these SNP subsets were significantly highly correlated (0.75 ≤ *r* ≤ 1) with those of the full SNP set for SNP sets larger than 1K SNPs and well correlated (0.38 ≤ *r* ≤ 0.84) for the smallest set of 100 SNPs (Table 1). Similarly, the accuracy ranged from 0.94 to 1 for SNP sets larger than 1K SNPs, with 0.8 being the lowest performance observed when analysing the smallest SNP set (Fig. 5A), demonstrating that even using 100 SNPs, the classification of an individual’s genetic similarity to a continental group remained robust.

**Table 1.** Correlation table for 1000 Genomes samples and various SNP subsets using the original cohort.

SNP set	Africa A	Africa B	Central Asia	South East Asia	India	Central Europe	South Europe	West Europe	Scandinavia	East Asia	Far East Asia	Native America
50K	1 (0.00E+00)	1 (0.00E+00)	0.99 (0.00E+00)	0.98 (0.00E+00)	1 (0.00E+00)	0.99 (0.00E+00)	0.99 (0.00E+00)	0.99 (0.00E+00)	0.99 (0.00E+00)	0.99 (0.00E+00)	0.99 (0.00E+00)	0.99 (0.00E+00)
10K	0.99 (0.00E+00)	0.99 (0.00E+00)	0.95 (0.00E+00)	0.93 (0.00E+00)	0.98 (0.00E+00)	0.97 (0.00E+00)	0.97 (0.00E+00)	0.94 (0.00E+00)	0.94 (0.00E+00)	0.96 (0.00E+00)	0.95 (0.00E+00)	0.93 (0.00E+00)
1K	0.92 (0.00E+00)	0.95 (0.00E+00)	0.84 (7.20E−256)	0.75 (3.22E−185)	0.92 (0.00E+00)	0.85 (7.85E−266)	0.89 (0.00E+00)	0.8 (1.57E−221)	0.8 (1.29E−220)	0.78 (2.13E−209)	0.83 (1.69E−253)	0.81 (1.44E−230)
100	0.7 (2.20E−161)	0.84 (2.29E−256)	0.38 (1.42E−45)	0.47 (1.27E−68)	0.56 (2.98E−97)	0.49 (2.19E−73)	0.56 (5.35E−97)	0.48 (2.65E−71)	0.56 (2.00E−95)	0.55 (3.26E−94)	0.56 (8.86E−99)	0.47 (4.20E−68)

The table shows the correlation (*r*) and (*P*-values (Student’s *t*-test)) between the genetic similarity to different continental groups (columns) of the original cohort calculated using the full SNP set and random subsets of various sizes (rows).

Next, we created an unseen dataset from the remaining 1829 (out of 2504 samples, 73%) 1000 Genomes samples that were not used to design the gene pools and the same SNPs. We applied *AncestryGeni* to this dataset. The genomic components calculated for these SNP subsets showed strong correlations (0.42 ≤ *r* ≤ 1) with those from the full SNP set (Table 2) when the subsets contained more than 1K SNPs. Even for the smallest SNP set, the correlations remained moderate to strong (0.23 ≤ *r* ≤ 0.82). Similarly, the accuracy ranged from 0.94 to 0.98 for SNP sets larger than 1K SNPs, with the lowest accuracy observed again when analysing the smallest SNP set (0.69–0.75) (Fig. 5B). Similar results were obtained for two dozen similar runs (results not shown). The high accuracy is reported across the three continental groups, demonstrating that the chosen SNPs, the gene pool model, and the SML algorithm can classify samples into the three continental groups for relatively homogeneous populations over small random SNP sets with high accuracy.

**Table 2.** Correlation table for 1000 Genomes samples and various SNP subsets using a new cohort.

SNP set	Africa A	Africa B	Central Asia	South East Asia	India	Central Europe	South Europe	West Europe	Scandinavia	East Asia	Far East Asia	Native America
50K	1 (0.00E+00)	1 (0.00E+00)	0.97 (0.00E+00)	0.95 (0.00E+00)	1 (0.00E+00)	0.98 (0.00E+00)	0.99 (0.00E+00)	0.97 (0.00E+00)	0.97 (0.00E+00)	0.98 (0.00E+00)	0.98 (0.00E+00)	0.96 (0.00E+00)
10K	0.96 (0.00E+00)	0.98 (0.00E+00)	0.62 (3.97E−119)	0.8 (3.20E−226)	0.98 (0.00E+00)	0.94 (0.00E+00)	0.56 (1.44E−95)	0.9 (0.00E+00)	0.9 (0.00E+00)	0.96 (0.00E+00)	0.94 (0.00E+00)	0.85 (5.67E−267)
1K	0.91 (0.00E+00)	0.95 (0.00E+00)	0.68 (6.64E−150)	0.42 (1.63E−53)	0.9 (0.00E+00)	0.78 (1.27E−210)	0.82 (2.01E−243)	0.59 (6.86E−107)	0.57 (2.61E−99)	0.66 (6.55E−140)	0.78 (6.17E−210)	0.65 (5.30E−134)
100	0.6 (3.82E−113)	0.82 (2.26E−242)	0.23 (1.08E−17)	0.23 (1.70E−17)	0.59 (8.47E−107)	0.38 (8.61E−44)	0.5 (9.29E−78)	0.34 (3.63E−35)	0.41 (6.20E−51)	0.49 (9.28E−75)	0.52 (4.80E−84)	0.28 (3.68E−24)

The table shows the correlation (*r*) and (*P*-values (Student’s *t*-test)) between the genetic similarity to different continental groups (columns) of a new cohort calculated using the full SNP set and random subsets of various lengths (rows).

### 3.4 *AncestryGeni* accurately classifies continental groups for the CoMMpass MM WES data

Next, we applied *AncestryGeni* to estimate an individual’s genetic similarity to the three continental groups available in the CoMMpass MM dataset (*n *= 448), where a mix of mutation callers were used to call SNPs from germline or somatic mutations from MM tumors. MM tumors were chosen as a real-world dataset because newly diagnosed MM typically exhibit around 7000 single nucleotide variants (SNVs) across the genome (Samur *et al.* 2020), along with evidence of hyper-APOBEC activity (Maura *et al.* 2024). We trained the model on one dataset at a time by randomly selecting 50% of its samples and testing the classification accuracy on all the datasets for samples not used for training. Whereas, the 1000 Genomes dataset was applied with the full SNP set of over 300 000 SNPs, the CoMMpass datasets had far fewer SNPs, specifically Strelka2 (germline) with a mean of 84 992 and standard deviation of 26 068 SNPs, HaplotypeCaller (germline) with 12 933 ± 3698 SNPs, and VarDict (somatic) with 14 449 ± 4037 SNPs. The classification accuracy ranged from 0.73 to 0.94, with Strelka2 (germline), when used in the testing dataset, showing the lowest consistency with the other two callers, compared to HaplotypeCaller 0.95–1 and VarDict 0.93–1, when used in the training dataset (Fig. 5C). By contrast, Strelka2 (germline), when used in the training dataset, showed high concordance in detecting the continental group genetic similarity of the CoMMpass WES data with high accuracy (0.77–0.95) but with a lower accuracy (0.68) when applied to the RNA-Seq dataset. The best-performing training dataset, the 1000 Genomes, allowed *AncestryGeni* to accurately (0.93–1) classify nearly all samples to the correct continental groups for data generated from the germline and somatic mutation calling tools (Fig. 5C). However, when applied to the RNA-Seq data (2136 ± 787 SNPs), this dataset had the weakest performance (0.61) compared to the other datasets, 0.64–0.91. When *AncestryGeni* was trained on the CoMMpass data, it achieved more accurate results. Importantly, training on the RNA-Seq dataset allowed sampling classification into the continental groups with the highest accuracy (0.91) when testing the RNA-Seq data (Fig. 5C).

These results show that somatic and germline mutations exhibit genotype differences that reduce the classification accuracy when training and testing on one another. Training on the 1000 Genomes dataset yielded the most robust classifications, except for the RNA-Seq data. However, it was possible to accurately classify the continental groups from the RNA-Seq data using the RNA-Seq as a training dataset. Further, since the results from the RNA-Seq data were in high congruence (0.64–0.68) with the results from the two germline and somatic callers, we hypothesized that the relatively homogeneous populations of the 1000 Genomes calculated over many SNPs may not be a suitable training dataset for classifying the continental group when analysed on a small number of SNPs.

### 3.5 *AncestryGeni* accurately classifies the continent groups for the CoMMpass MM RNA-Seq data

To test whether *AncestryGeni* is suitable for RNA-Seq data, we randomly selected 1200 SNPs from each dataset and repeated the above calculation, using one dataset for training and the remaining datasets for testing. We found that the strong performances of *AncestryGeni* (Fig. 5C) were improved (Fig. 5D) using this smaller SNP set.

Training on the 1000 Genomes dataset still yielded highly accurate results for all datasets (0.79–0.97), except for RNA-Seq (0.41), in general agreement with the results obtained for the complete SNP set (Fig. 5C), which may reflect the mismatch between the relatively homogeneous 1000 Genomes samples and the more heterogeneous CoMMpass populations. By contrast, the classification accuracy of all the mutation callers used for the CoMMpass data ranged from 0.91 to 0.99, eliminating the previously observed differences between somatic and germline callers for WES. Importantly, all these tools could classify the continental group using the RNA-Seq data with very high accuracy (0.75–0.84) when using the RNA-Seq dataset as the testing dataset. Interestingly, RNA-Seq data was the best classifier of the continental group for itself (0.91) and somatic and germline callers (0.92–0.97).

**Figure 6.**
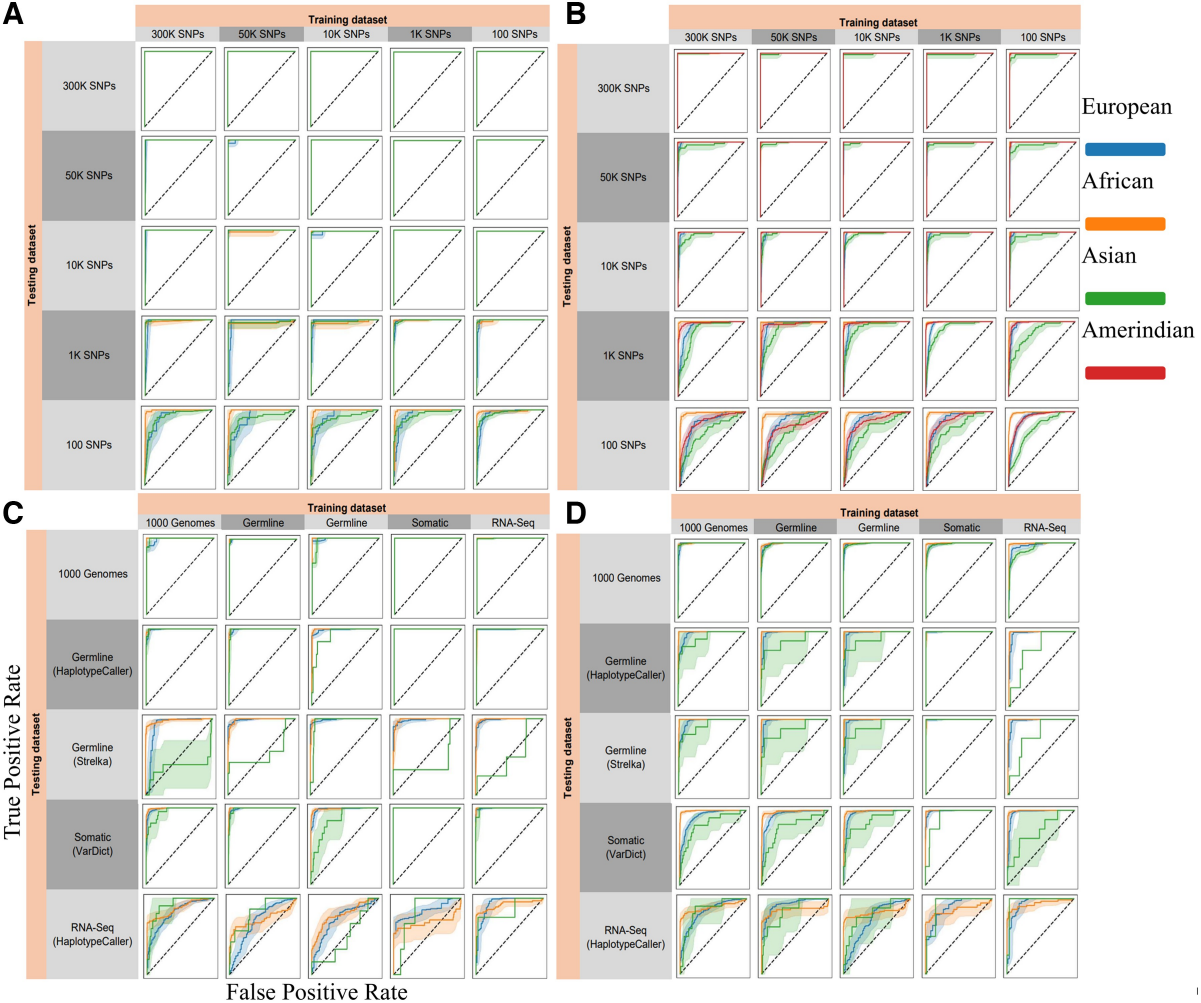
ROC curves evaluate the performance of *AncestryGeni* using the same dataset configurations as Fig. 5. The curves show the True Positive Rate against the False Positive Rate, with the diagonal dashed line representing random classification performance. Excellent classification is indicated by curves reaching the upper-left corner, as seen in panels with larger SNP sets (300K–1K). ROC curves were computed in a multi-class setting using a one-vs-rest (OvR) strategy, where each ancestry group was evaluated against all others independently. (A, B) Performance evaluation across varying SNP panel sizes shows that AncestryGeni maintained discriminative power even with reduced SNP counts, with increasing uncertainty in 100 SNP panels (indicated by broader confidence intervals). (C, D) Cross-dataset evaluation using different genomic inputs demonstrates robust classification across data types, with some variation in performance particularly evident in RNA-Seq classifications. Population-specific classification power is shown by colored lines, with shaded bands representing 95% confidence intervals derived from 1000 bootstrap iterations. The sample sizes for each figure are as follows: For all subplots in (A), we used 460 samples for the training set and 115 samples for all the test experiments. For all subplots in (B), the training set consisted of 1463 samples, and 366 samples were used for all the test experiments. In C, the training set included 212 samples, and the test experiments used 363 samples for 1000 Genomes, 443 for Germline HaplotypeCaller, 447 for Germline Strelka2, 448 for RNA-Seq, and 878 for Somatic Verdict. For D, the training set consisted of 176 samples, and the test experiments included 575 samples for 1000 Genomes 1K, 272 for Germline HaplotypeCaller 1K, 272 for Germline Strelka2 1K, 272 for RNA-Seq, and 544 for Somatic Verdict 1K.

**Figure 7.**
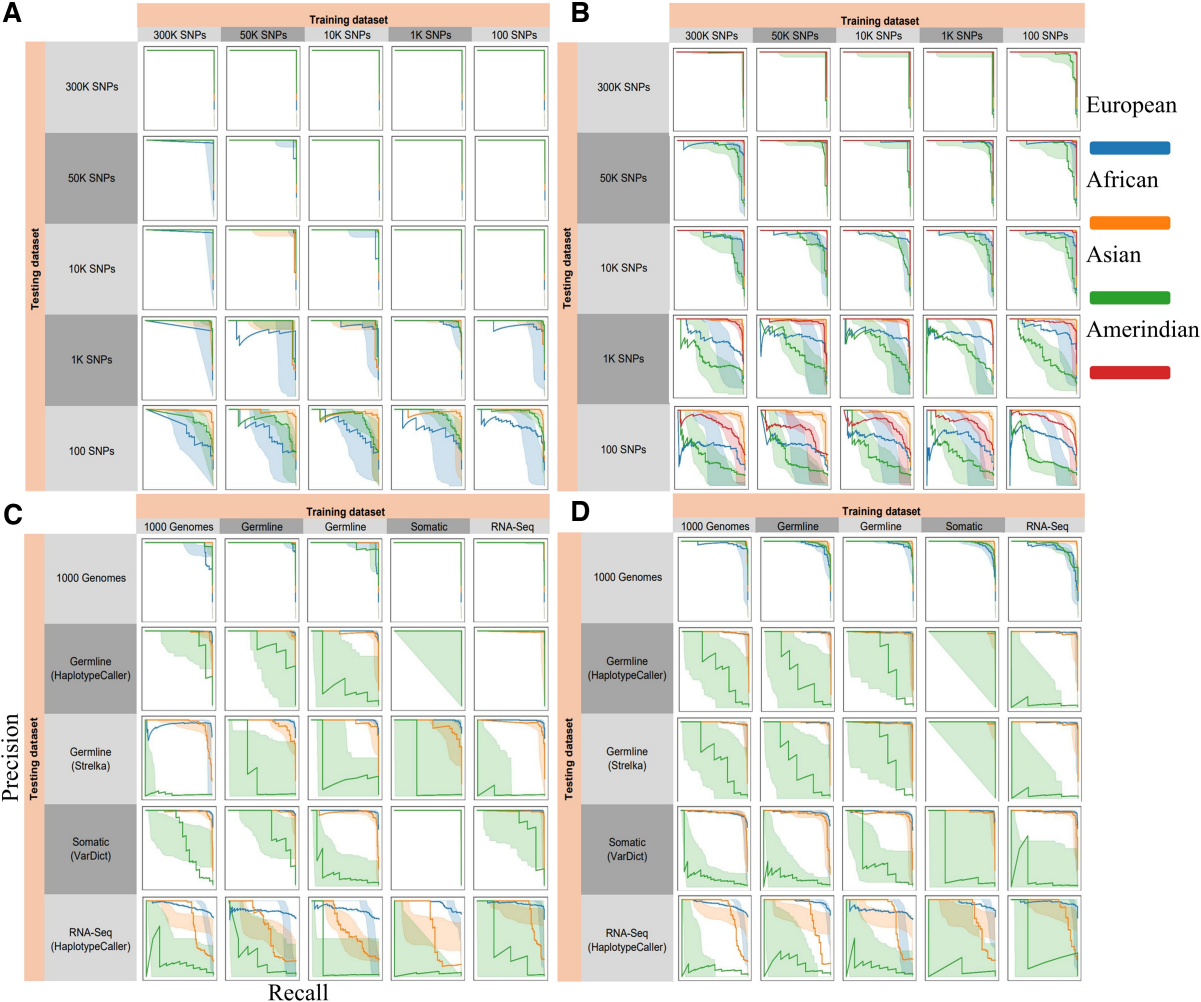
Precision–recall curves for *AncestryGeni* performance evaluation using the same dataset configuration as Fig. 5. The curves illustrate the relationship between precision (positive predictive value) and recall (sensitivity), where curves approaching the upper-right corner indicate optimal performance. Multi-class performance was assessed using a one-vs-rest (OvR) strategy, where precision and recall were calculated independently for each ancestry group against the rest. (A, B) SNP panel analysis demonstrates high precision–recall values maintained across ≥1K SNPs, with visible performance stratification at 100 SNPs, particularly pronounced in cross-population classifications. (C, D) Genomic input evaluation reveals data type-specific performance patterns, with RNA-Seq showing distinct precision–recall tradeoffs compared to DNA-based inputs. Population-specific performance is indicated by colored lines, with shaded bands representing 95% confidence intervals derived from 1000 bootstrap iterations. Perfect classification is characterized by curves maintaining high precision across all recall values, as observed in the high-SNP panels.

To further evaluate *AncestryGeni’s* performance across different SNP densities and regional populations, we generated ROC curves (Fig. 6) (Green and Swets 1966) in a multi-class setting using a OvR strategy, where each ancestry group was evaluated against all others independently and precision–recall curves (Fig. 7) (Davis and Goadrich 2006) using the same dataset configurations described above. As previously observed (e.g., Fig. 5), at high SNP densities (50K–300K), the areas under the curve (AUC) approached 1.0 for all populations (Fig. 6A and B) and the corresponding precision–recall curves maintained high values in both metrics, particularly for European and African populations (Fig. 7A and B). As SNP density decreased to 10K, subtle degradation appeared in both ROC and precision–recall curves, especially for Asian and Amerindian populations, a likely reflection of the small sample size for these populations. We observed decreased ROC and precision–recall curve performance at lower densities (1K and 100 SNPs). When evaluating different genetic data types, performance on 1000 Genomes data remained robust, however notable declines were observed for germline, somatic, and RNA-Seq data, with RNA-Seq showing the most significant performance degradation, particularly evident in the stepped patterns of its precision–recall curves (Fig. 7C and D).

### 3.6 *AncestryGeni* enables accurate regional classification for genetically diverse individuals using refined genetic mapping

The classification process was based on a dataset derived from the Human Genome Diversity Project (HGDP), containing genetically diverse individuals. To ensure accurate regional assignments, data processing was conducted in two key steps: In the first step, regional labels were systematically refined to resolve ambiguities and improve classification precision. For instance, populations within Asia were separated into two subgroups: East Asia and Central Asia. This distinction was based on known population-level genetic characteristics and geographic origins. Specific populations such as Han, Japanese, and Tujia were reassigned to East Asia, while others remained under Central Asia. Smaller regions, including North Africa and Sub-Saharan Africa, were grouped into the broader category of Africa. These refinements provided a logical framework for consistent labeling across all samples, aligning regional classifications with known genetic and geographic relationships.

In the second step, the dataset was normalized to ensure compatibility with machine learning models. Ancestral proportions, represented as feature columns, were scaled proportionally to eliminate noise and ensure consistent contribution from all features. During cross-validation, a stratified sampling strategy was employed to maintain representative distributions across all population groups, preventing bias in model training and evaluation. To test which SML model can best classify individuals into the continental groups from the genomic components, we evaluated the most common SML algorithms, including, but not limited to, LDA, ridge classifier, logistic regression, random forest, gradient boosting, and naive Bayes. Because the LDA algorithm outperformed all the other SMLs (Table 3), we adopted it to detect the continental groups. An LDA model was subsequently trained on the processed data. Evaluation metrics, including accuracy, recall, precision, *F*1-score, and AUC, confirmed the model’s reliability. By combining precise regional mapping with rigorous preprocessing and stratified cross-validation, the pipeline achieved high classification performance, demonstrating its effectiveness in ancestry identification.

**Table 3.** Ancestry classification with LDA on 1000 Genomes Data.

Model	Accuracy	AUC	Recall	Precision	*F*1	Kappa	MCC	TT (s)
Linear discriminant analysis (LDA)	0.97	0.999	0.97	0.974	0.97	0.959	0.96	0.006
Naive Bayes	0.97	0.996	0.97	0.974	0.971	0.959	0.96	0.006
SVM—Linear Kernel	0.963	1	0.963	0.969	0.962	0.948	0.95	0.032
Ridge classifier	0.941	NaN	0.941	0.955	0.937	0.917	0.922	0.004
Logistic regression	0.941	0.999	0.941	0.955	0.937	0.917	0.922	0.008
Light gradient boosting machine	0.941	0.989	0.941	0.942	0.938	0.917	0.918	0.168
Ada boost classifier	0.933	0.797	0.933	0.948	0.934	0.908	0.911	0.065
Random forest classifier	0.919	0.978	0.919	0.929	0.906	0.884	0.891	0.114
Extra trees classifier	0.911	0.988	0.911	0.914	0.899	0.874	0.88	0.118
Extreme gradient boosting	0.889	0.986	0.889	0.905	0.883	0.844	0.85	0.177
Gradient boosting classifier	0.844	0.973	0.844	0.839	0.834	0.779	0.785	0.38
*K* neighbors classifier	0.8	0.953	0.8	0.785	0.789	0.718	0.72	0.01
Decision tree classifier	0.726	0.796	0.726	0.735	0.726	0.625	0.629	0.007
Quadratic discriminant analysis	0.644	0.62	0.644	0.804	0.644	0.497	0.565	0.006

Using 10-fold cross-validation on the 1000 Genomes Project dataset, with 1K SNPs as training data and 300K SNPs as testing data, LDA emerged as the most suitable choice due to its superior accuracy (97%), high AUC (99%), and low training time (0.006 s). These results demonstrated that LDA could efficiently classify genetic ancestry data while balancing computational speed and performance.

**Figure 8.**
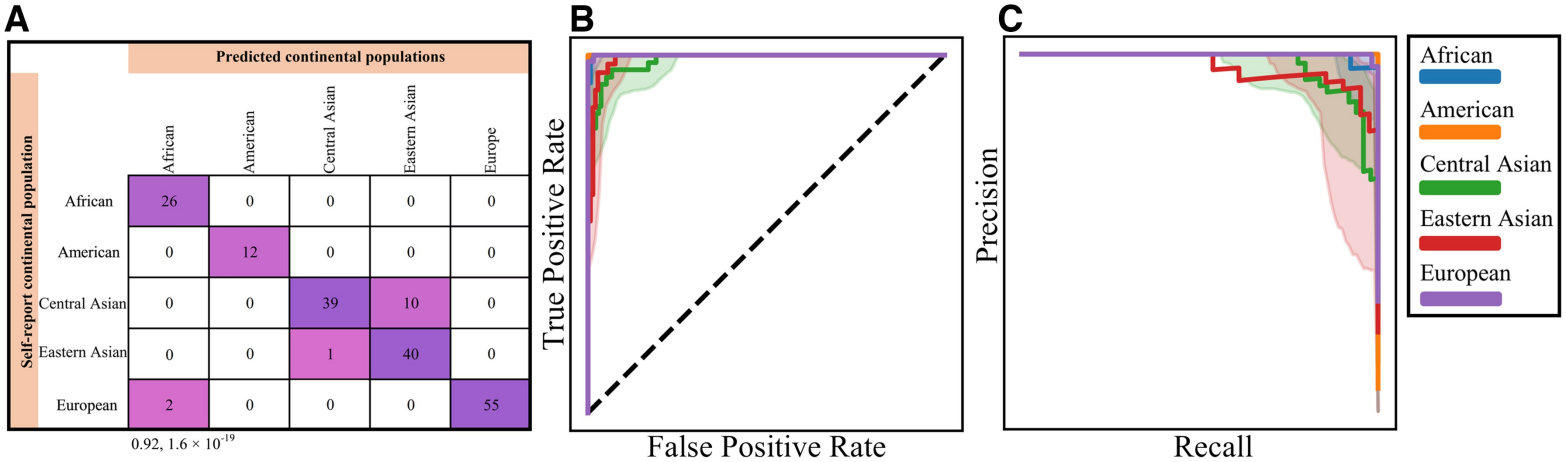
Performance evaluation of LDA model for HGDP mixed individual classification. (A) A confusion matrix displaying classification outcomes across five continental groups (African, American, Central Asian, Eastern Asian, and European). The diagonal values represent correct classification, showing strong performance. (B) The ROC curve demonstrates the model’s ability to distinguish between classes, performing well above the random classifier baseline (dashed line), indicating strong discriminative capability. (C) A precision–recall plot showing the tradeoff between precision and recall for each continental group, with varying performance across different populations—notably strong performance for European and Eastern Asian groups as indicated by the higher curves. Accuracy and *P*-value are shown under the confusion matrix.

Overall, *AncestryGeni* demonstrated robust performance across all the continental groups, achieving an overall accuracy of 0.92 (Fig. 8A). The confusion matrix (Fig. 8A) showed that European and Eastern Asian populations had the highest number of correct classifications, with some cross-classification observed between Central and Eastern Asian populations. The classification accuracy for Americans and Africans ranged from very high to perfect. The ROC curves (Fig. 8B) indicate exceptional discriminative ability with AUC values of 1.00 for Africans, Americans, and Europeans and 0.99 for both Central and Eastern Asian groups. The precision–recall curves (Fig. 8C) further validate the tool’s robust classification capability across all the continental groups, particularly for European and Eastern Asian ancestries. Additionally, classification metrics such as *F*1-score (0.93), Kappa (0.91), and MCC (0.91) emphasize the model’s consistency and reliability across all evaluated populations.

For WGS data (*n *= 471), *AncestryGeni* took 5–48 min to complete (for 10%–100% samples) at a similar memory usage of 230–250 MB. For RNA-Seq data (*n *= 478), it took only 1–10 min to complete with a memory usage of 33–34 MB. For both datasets, run time increased linearly following the increase in sample size.

### 3.7 *AncestryGeni* accurately classifies perturbed RNA-Seq data

**Figure 9.**
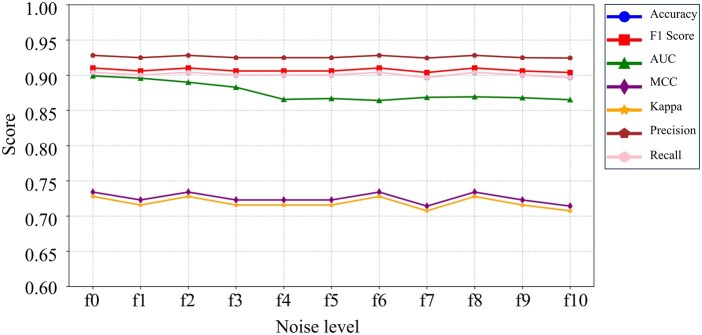
*AncestryGeni* classification performance under perturbation. Model performance evaluation metrics across increasing perturbation levels (f0–f9) on a balanced dataset of 271 samples per perturbation level. Performance metrics include accuracy, *F*1 score, ROC-AUC (one-vs-rest), MCC, Cohen’s Kappa, precision, and recall. Results demonstrate robust model stability, with upper-band metrics (accuracy, *F*1, precision, recall) maintaining scores above 0.90 and AUC above 0.85, while correlation metrics (MCC, Kappa) show consistent performance around 0.72 across all perturbation levels.

**Figure 10.**
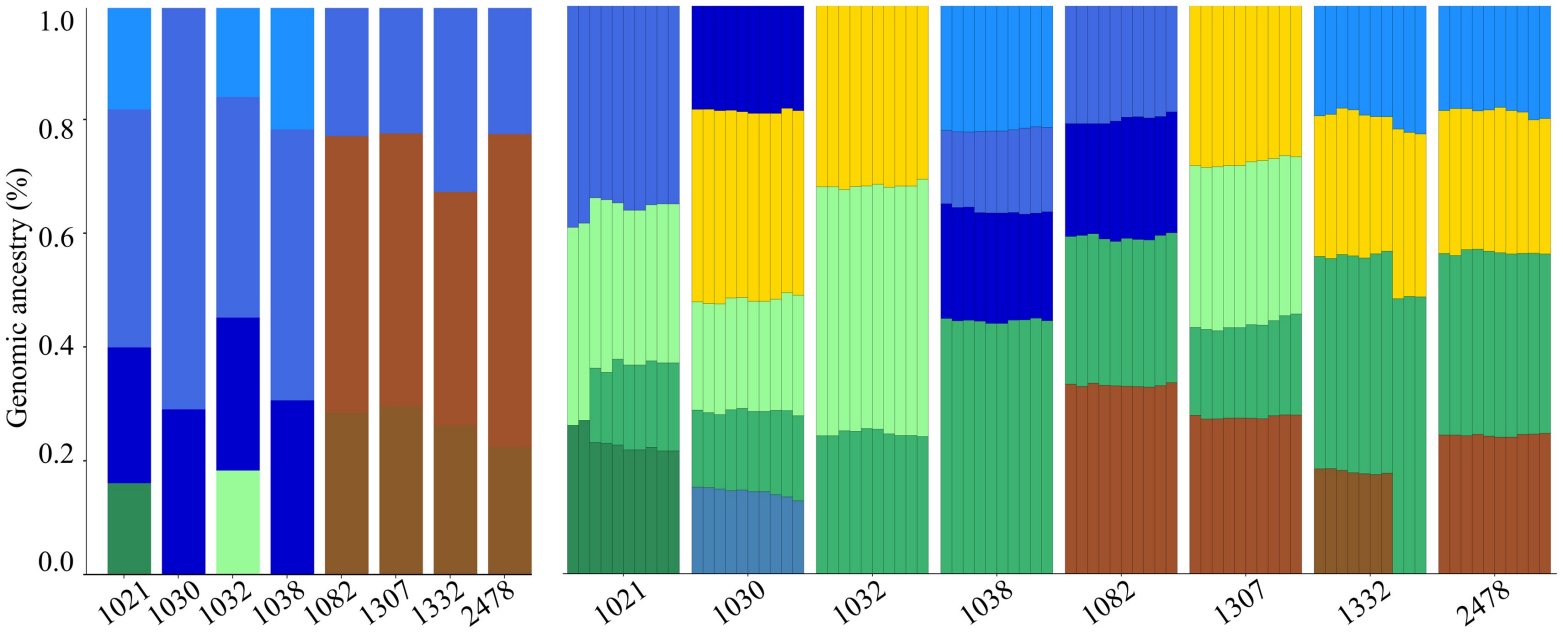
Admixture proportions of eight random samples in a noisy environment of RNA-Seq data. Left panel shows baseline genomic ancestry components for eight individuals (CoMMpass samples) derived from germline variant calling (HaplotypeCaller). Right panel demonstrates the stability of these ancestry estimates under increasing noise levels (f0–f9) using RNA-Seq data. Each vertical bar represents an individual’s ancestry proportions, with colors indicating different ancestral populations: African (brown), Asian/European (green shades), Native American (yellow), and European (blue shades). Components contributing less than 0.1 were removed, and the proportions were renormalized. In the right panel, each individual is represented by 10 adjacent bars corresponding to different noise levels, demonstrating the robustness of ancestry estimation across varying data quality conditions.

DNA alterations may arise biologically through somatic tumor mutations or are introduced artificially due to technical noise in the data. To assess *AncestryGeni’s* performance under systematic perturbation, we introduced perturbations with increasing levels from f0 (baseline) to f9 (maximum noise) to the most challenging dataset of RNA-Seq, where the number of SNPs ranged from 627 to 6592, with a median of 2009. We then evaluated classification accuracy (Fig. 9). Despite the perturbations (Fig. 10), the model correctly classified the individuals to their continental group with high accuracy (0.92). Moreover, the accuracy remained stable across all noise levels as measured by seven matrices, indicating the robustness of the model to systematic perturbations, which affected the variation within the genetic components but did not modify them. These findings support using *AncestryGeni* to evaluate tumor samples with high tumor mutation burdens.

### 3.8 *AncestryGeni* reliably classifies synthetic admixture and captures underlying dual ancestry signals

**Figure 11.**
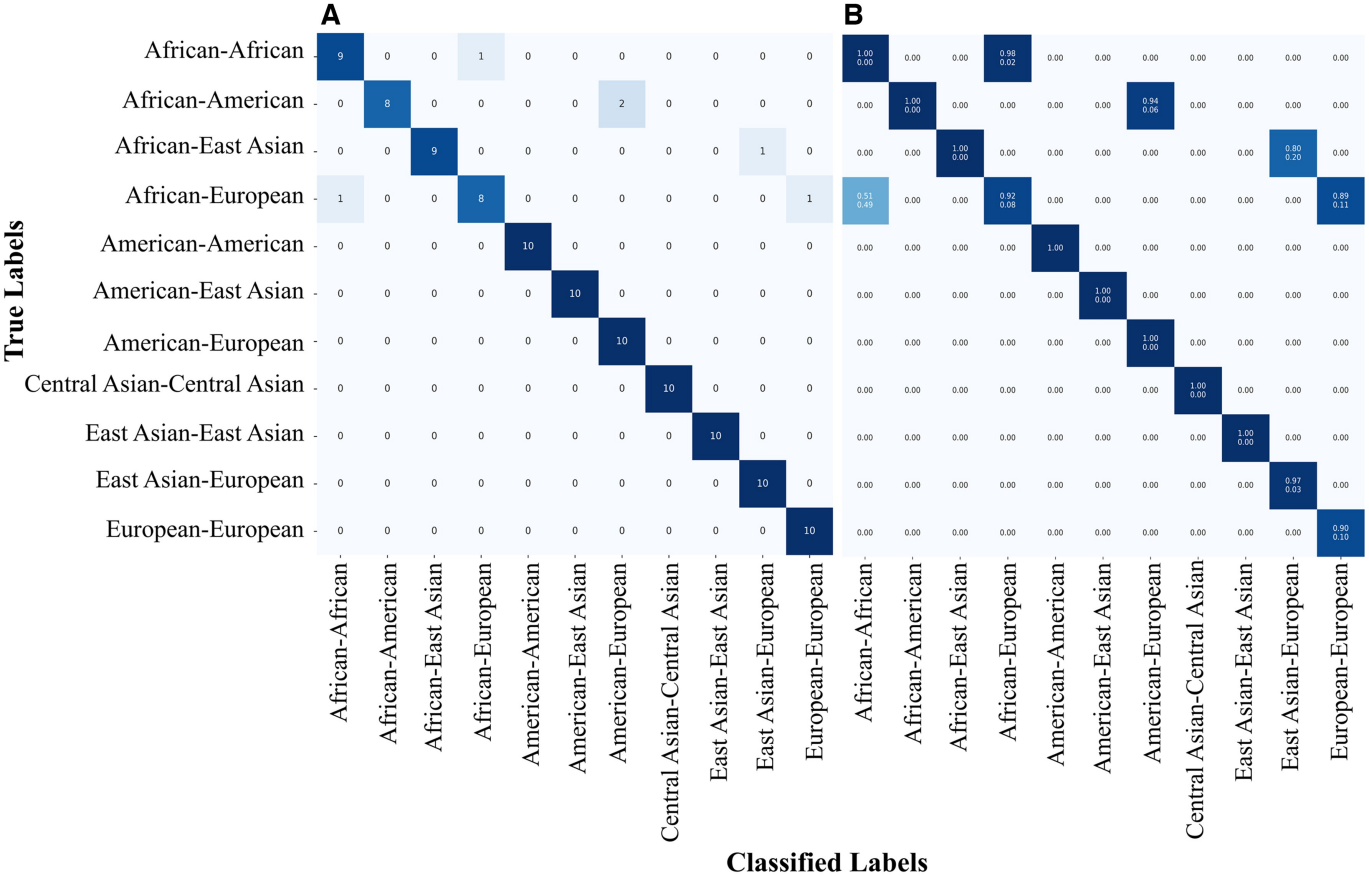
LDA classification performance on synthetic admixed individuals. (A) Confusion matrix displaying the final labels for test samples, showing perfect classification across all 11 ancestry categories. (B) Probability matrix indicating the average top-1 and top-2 confidence scores per true-classified label pair. While the confusion matrix shows full accuracy for categories like *European-European*, the corresponding probability heatmap reveals a lower average confidence (∼0.90), highlighting the model’s internal uncertainty despite correct classification. This reflects subtle overlaps in genomic components, especially for admixed groups.

To assess classification robustness for admixture, synthetic samples were generated by averaging ancestry components from paired continental groups. *AncestryGeni* achieved an overall accuracy of 94.55% across 11 admixed ancestry groups (*n *= 10 samples per group) comprising both homogeneous and admixed ancestry types, with seven groups achieving a perfect classification (10/10) (Fig. 11A). Misclassifications were concentrated in four admixed African groups, demonstrating that misclassifications largely occurred due to heterogeneous ancestry, with highly mixed samples being correctly assigned to more than two source populations (Table 4). *AncestrGeni* also provides the classification probabilities, quantifying the model’s confidence in its classifications (Fig. 11B). Individuals sampled from the homogeneous ancestry groups received high confidence scores, whereas individuals of mixed ancestries exhibit nuanced probabilities reflecting their multi-ancestral origin. This modeling approach demonstrates that even with simple linear methods, *AncestrGeni* accurately classifies highly mixed individuals to their source populations.

**Table 4.** Low confidence classification outcomes for selected test individuals with top-1 probabilities below 0.85.

Sample_Name	True ancestry	Predicted ancestry	Top1 results	Top1 probability	Top2 results	Top2 probability
HGDP00635_HGDP00670	Africa-Europe	Africa-Europe	Africa-Europe	0.863	Africa-Africa	0.137
HGDP00802_HGDP00759	Africa-East Asia	East Asia-Europe	East Asia-Europe	0.802	Africa-East Asia	0.198
HGDP00797_HGDP00370	Africa-Europe	Africa-Europe	Africa-Europe	0.711	Europe-Europe	0.289
HGDP00659	Europe-Europe	Europe-Europe	Europe-Europe	0.649	Africa-Europe	0.351
HGDP00336	Europe-Europe	Europe-Europe	Europe-Europe	0.554	Africa-Europe	0.446
HGDP00566_HGDP00335	Africa-Europe	Africa-Africa	Africa-Africa	0.51	Africa-Europe	0.49

These six samples include both synthetic admixed individuals (created by averaging gene pools from two HGDP samples) and real individuals from the Europe-Europe group. While most results aligned with the true ancestry labels, their low confidence values indicate ambiguity in ancestry boundaries. Top-2 probabilities often reflected biologically plausible secondary affiliations—such as Africa-Africa or Europe-Europe—highlighting historical admixture signals. Probabilities are reported as decimal values (e.g. 0.863 corresponds to 86.3% model confidence).

### 3.9 Limitations

While our data indicate that a high somatic mutation burden—such as that observed in MM—or technical noise does not substantially compromise the accuracy of ancestry inference, we cannot exclude the possibility that exceptionally high mutation loads seen in some solid tumors, like lung or melanoma (Martincorena and Campbell 2015), may influence ancestry assignment. Additionally, we demonstrated that *AncestryGeni* is capable of handling individuals with two-way admixture. However, in individuals with greater or more complex admixture patterns, the continental groups typically reflect only the dominant ancestry components, rather than all contributing regions. In such cases, gene pool results and assignment probabilities that capture the complexity of their familial history provide a more accurate representation.

## 4 Discussion

The rapid adoption of genomic sequencing technologies has increased the use of genetic ancestry data in health disparity research. Genetic similarity to different continental groups can be inferred from AIMs extracted from WGS and SNP arrays of genomic DNA (Carress *et al.* 2021). While germline DNA is ideal for estimating ancestry, tumor-derived WES or RNA-Seq are often the only available data sources. Consequently, ∼90% of clinical laboratories perform tumor-only NGS studies without a matching germline sample (Hunt *et al.* 2022). The use of these tumor-derived sources, particularly RNA-Seq, poses challenges in inferring genetic ancestry. The alternatives of using self-reported race or inaccurate algorithms are disadvantageous.

Inaccurate algorithms are only one outcome of built-in inequalities in the health system. AA individuals and many other minorities are underrepresented in most research studies, limiting our understanding of the mechanisms behind health disparities and preventing us from developing inclusive advanced computational tools as we progress beyond inaccurate measures like principal component analysis (PCA) (Elhaik 2022) and leverage the most recent advances in computational biology. SML based tools have successfully been implemented across health systems, yet it remains unclear how to train them. Inspired by previous studies (Elhaik *et al.* 2014, Elhaik and Ryan 2019), we developed an SML that is applied to the sample’s genetic ancestry, i.e., the autosomally-inherited DNA lineage of the individual, represented as a “profile” of the proportions of regional groups where their alleles are the most common.

Thus, the value of this study is the development of a novel SML tool designed to infer genetic ancestry from tumor-derived sequencing data, such as WES or RNA-Seq, which often lack matching germline DNA. In cancer research, germline samples can be unavailable, limiting the ability to study ancestry-related biology. This tool addresses that gap by leveraging available tumor sequencing data to infer ancestry accurately when ideal germline references are not accessible. The gene pool data, a direct output from the algorithm (see Supplementary Note, available as supplementary data at *Bioinformatics* online), can be applied as a continuous variable in health equity research and is used as input to classify samples into continental groups, which can be used as a categorical variable. An insufficient number of markers is also a prohibitive factor when choosing the best analytical tool, which has limited ancestry inference in RNA-Seq, due to the variable nature of the markers and their small number. We demonstrated that *AncestryGeni* produces accurate results with as few as 100 SNPs (Fig. 5). We obtained 2137 SNPs on average (with a range of 630–6700 SNPs) for individuals in the CoMMpass study, which generated very high coverage data of 200 million read pairs (on average). Therefore, even using low coverage (20 million SNPs) may be sufficient to employ *AncestryGeni* for over 95% of the samples.

The biological interpretation of ancestry results, particularly for admixed populations, is crucial for understanding health disparities and implementing precision medicine approaches. Individuals sharing genetic similarities will exhibit similar profiles and similar genetic histories, which can be used to find their geographic origins and optimize their classification into cases and controls (Elhaik *et al.* 2014, Elhaik and Ryan 2019). Populations with genetically diverse backgrounds who have ancestry from more than one genetic group can also be analysed under this model, though the interpretation of their geographic origins should be done with models that consider multiple origins (e.g. GPS Origins by HomeDNA, https://homedna.com/product/gps-origins) rather than a single origin (e.g., Elhaik et al.’s GPS). These inferences can influence many aspects of human health, where ancestry plays a critical role in assessing risk for various conditions and diseases, like the high dispositions of AAs for MM and its precursor condition, MGUS, compared to Europeans. Other examples include a predisposition to inherited diseases, like Tay-Sachs or Gaucher (Type 1) diseases among Ashkenazic Jews, G6PD Deficiency among AA, and Alpha-Thalassemia among Southeast Asians. Because genetic screening panels are typically designed to target a limited number of well-characterized variants that are highly prevalent in specific populations, and universal panels, while more inclusive, may still fail to capture rare or population-specific variants, particularly those found in underrepresented or genetically distinct groups—the wrong choice of panel can lead to missed diagnoses or incomplete risk assessments, especially in individuals with ancestry from populations where certain variants are rare globally, but more common locally.

Targeted screening, informed by a patient’s ancestry, largely improves the likelihood of detecting clinically relevant variants. The importance of ancestry inference on health disparities, coupled with the limitation of current methods to process RNA-Seq data, underscores the need for a new all-in-one classifier like *AncestryGeni*. We showed that *AncestryGeni* can accurately estimate an individual’s genetic ancestry and is applicable to various genomic data types, including RNA-Seq data, from healthy and tumor tissues, often the only available genomic source in clinical settings. We note that *AncestryGeni* is not limited to classifying the continental groups and can be calibrated to identify any number of regions, depending on the training set (e.g., Fig. 8). We also acknowledge that although the somatic tumor mutation burden found in MM or technical noise does not appear to compromise the accuracy of ancestry inference, we cannot entirely rule out the possibility that an exceptionally high tumor mutation burden may influence gene pool estimates. Further studies using multiple tumor types beyond MM are warranted to address this potential limitation. *AncestryGeni* outperforms existing methods like FastNGSadmix, especially when using nonstandard genomic material and RNA-Seq data. We also showed that *AncestryGeni* performs well even for as few as 100 SNPs and is agnostic to the mutation caller tool. The ability to analyse perturbed genomic DNA using *AncestryGeni* may increase the inclusion of genomic data from underrepresented populations, particularly in the absence of self-reported race data.

## Supplementary Material

btaf391_Supplementary_Data

## Data Availability

CoMMpass data used in this study are publicly available [database of Genotypes and Phenotypes (dbGap): phs000748.v1.p1 and EGAS00001001178] and also at http://www.ncbi.nlm.nih.gov/bioproject/248538. The MGP1000 pipeline is freely and publicly available at https://github.com/pblaney/mgp1000.* AncestryGeni* pipeline is available at https://github.com/eelhaik/AncestryGeni/tree/main. Detailed instructions on file structure, commands, and performances are in **Supplementary Note**.

## References

[btaf391-B1] Alexander DH , Lange K. Enhancements to the ADMIXTURE algorithm for individual ancestry estimation. BMC Bioinformatics 2011;12:246. 21682921 10.1186/1471-2105-12-246 PMC3146885

[btaf391-B2] Baran Y , Pasaniuc B, Sankararaman S et al Fast and accurate inference of local ancestry in Latino populations. Bioinformatics 2012;28:1359–67. 22495753 10.1093/bioinformatics/bts144 PMC3348558

[btaf391-B3] Baughn LB , Li Z, Pearce K et al The CCND1 c.870G risk allele is enriched in individuals of African ancestry with plasma cell dyscrasias. Blood Cancer J 2020;10:39. 32179748 10.1038/s41408-020-0294-5 PMC7075993

[btaf391-B4] Baughn LB , Pearce K, Larson D et al Differences in genomic abnormalities among African individuals with monoclonal gammopathies using calculated ancestry. Blood Cancer J 2018;8:96. 30305608 10.1038/s41408-018-0132-1 PMC6180134

[btaf391-B5] Behnamian S , Esposito U, Holland G et al Temporal population structure, a genetic dating method for ancient Eurasian genomes from the past 10,000 years. Cell Rep Methods 2022;2:100270. 36046618 10.1016/j.crmeth.2022.100270 PMC9421539

[btaf391-B6] Bolger AM , Lohse M, Usadel B. Trimmomatic: a flexible trimmer for Illumina sequence data. Bioinformatics 2014;30:2114–20. 24695404 10.1093/bioinformatics/btu170 PMC4103590

[btaf391-B7] Bryc K , Durand EY, Macpherson JM et al The genetic ancestry of African Americans, Latinos, and European Americans across the United States. Am J Hum Genet 2015;96:37–53. 25529636 10.1016/j.ajhg.2014.11.010 PMC4289685

[btaf391-B8] Carress H , Lawson DJ, Elhaik E. Population genetic considerations for using biobanks as international resources in the pandemic era and beyond. BMC Genomics 2021;22:351. 34001009 10.1186/s12864-021-07618-x PMC8127217

[btaf391-B9] Coop G. Genetic similarity versus genetic ancestry groups as sample descriptors in human genetics. arXiv, 10.48550/arXiv.2207.11595, 2022, preprint: not peer reviewed.

[btaf391-B10] Davis J , Goadrich M. The relationship between precision–recall and ROC curves. In: *Proceedings of the 23rd International Conference on Machine Learning, 2006 June 25–29, Pittsburgh, PA, USA*. New York, NY, USA: Association for Computing Machinery*,* 2006.

[btaf391-B11] Elhaik E. Empirical distributions of F(ST) from large-scale human polymorphism data. PLoS One 2012;7:e49837. 23185452 10.1371/journal.pone.0049837 PMC3504095

[btaf391-B12] Elhaik E. Principal component analyses (PCA)-based findings in population genetic studies are highly biased and must be reevaluated. Sci Rep 2022;12:14683. 36038559 10.1038/s41598-022-14395-4 PMC9424212

[btaf391-B13] Elhaik E , Ryan DM. Pair Matcher (PaM): fast model-based optimization of treatment/case-control matches. Bioinformatics 2019;35:2243–50. 30445488 10.1093/bioinformatics/bty946 PMC6596890

[btaf391-B14] Elhaik E , Tatarinova T, Chebotarev D et al; Genographic Consortium. Geographic population structure analysis of worldwide human populations infers their biogeographical origins. Nat Commun 2014;5:3513. 24781250 10.1038/ncomms4513 PMC4007635

[btaf391-B15] Esposito U , Das R, Syed S et al Ancient ancestry informative markers for identifying fine-scale ancient population structure in Eurasians. Genes (Basel) 2018;9:1–18. 10.3390/genes9120625 PMC6316245 30545160

[btaf391-B16] Falush D , Stephens M, Pritchard JK. Inference of population structure using multilocus genotype data: linked loci and correlated allele frequencies. Genetics 2003;164:1567–87. 12930761 10.1093/genetics/164.4.1567 PMC1462648

[btaf391-B17] Fischer EM. Linear discriminant analysis. Stat Discrete Methods Data Sci 1936;392:1–5.

[btaf391-B18] Genomes Project C , Auton A, Brooks LD et al A global reference for human genetic variation. Nature 2015;526:68–74. 26432245 10.1038/nature15393 PMC4750478

[btaf391-B19] Graur D , Zheng Y, Price N et al On the immortality of television sets: "function" in the human genome according to the evolution-free gospel of ENCODE. Genome Biol Evol 2013;5:578–90. 23431001 10.1093/gbe/evt028 PMC3622293

[btaf391-B20] Green DM , Swets JA. Signal Detection Theory and Psychophysics. New York: Wiley, 1966, xi, 455.

[btaf391-B21] Greenberg AJ , Vachon CM, Rajkumar SV. Disparities in the prevalence, pathogenesis and progression of monoclonal gammopathy of undetermined significance and multiple myeloma between blacks and whites. Leukemia 2012;26:609–14. 22193966 10.1038/leu.2011.368 PMC3629947

[btaf391-B22] Hunt SE , Moore B, Amode RM et al Annotating and prioritizing genomic variants using the ensembl variant effect predictor – a tutorial. Hum Mutat 2022;43:986–97. 34816521 10.1002/humu.24298 PMC7613081

[btaf391-B23] Jorsboe E , Hanghoj K, Albrechtsen A. fastNGSadmix: admixture proportions and principal component analysis of a single NGS sample. Bioinformatics 2017;33:3148–50. 28957500 10.1093/bioinformatics/btx474

[btaf391-B24] Kim S , Scheffler K, Halpern AL et al Strelka2: fast and accurate calling of germline and somatic variants. Nat Methods 2018;15:591–4. 30013048 10.1038/s41592-018-0051-x

[btaf391-B25] Korneliussen TS , Albrechtsen A, Nielsen R. ANGSD: analysis of next generation sequencing data. BMC Bioinformatics 2014;15:356. 25420514 10.1186/s12859-014-0356-4 PMC4248462

[btaf391-B26] Lai Z , Markovets A, Ahdesmaki M et al VarDict: a novel and versatile variant caller for next-generation sequencing in cancer research. Nucleic Acids Res 2016;44:e108. 27060149 10.1093/nar/gkw227 PMC4914105

[btaf391-B27] Landgren O , Katzmann JA, Hsing AW et al Prevalence of monoclonal gammopathy of undetermined significance among men in Ghana. Mayo Clin Proc 2007;82:1468–73. 18053453 10.1016/S0025-6196(11)61089-6

[btaf391-B28] Landgren O , Kristinsson SY, Goldin LR et al Risk of plasma cell and lymphoproliferative disorders among 14621 first-degree relatives of 4458 patients with monoclonal gammopathy of undetermined significance in Sweden. Blood 2009;114:791–5. 19182202 10.1182/blood-2008-12-191676 PMC2716021

[btaf391-B29] Landgren O , Kyle RA, Pfeiffer RM et al Monoclonal gammopathy of undetermined significance (MGUS) consistently precedes multiple myeloma: a prospective study. Blood 2009;113:5412–7. 19179464 10.1182/blood-2008-12-194241 PMC2689042

[btaf391-B30] Lawson DJ , Hellenthal G, Myers S et al Inference of population structure using dense haplotype data. PLoS Genet 2012;8:e1002453. 22291602 10.1371/journal.pgen.1002453 PMC3266881

[btaf391-B31] Li H , Durbin R. Fast and accurate long-read alignment with Burrows–Wheeler transform. Bioinformatics 2010;26:589–95. 20080505 10.1093/bioinformatics/btp698 PMC2828108

[btaf391-B32] Martincorena I , Campbell PJ. Somatic mutation in cancer and normal cells. Science 2015;349:1483–9. 26404825 10.1126/science.aab4082

[btaf391-B33] Mathieson I , Scally A. What is ancestry? PLoS Genet 2020;16:e1008624. 32150538 10.1371/journal.pgen.1008624 PMC7082057

[btaf391-B34] Maura F , Rajanna AR, Ziccheddu B et al Genomic classification and individualized prognosis in multiple myeloma. J Clin Oncol 2024;42:1229–40. 38194610 10.1200/JCO.23.01277 PMC11095887

[btaf391-B35] National Academies of Sciences, Engineering, and Medicine. Using Population Descriptors in Genetics and Genomics Research: A New Framework for an Evolving Field. Washington, DC: The National Academies Press: 2023. 36989389

[btaf391-B36] Popejoy AB , Fullerton SM. Genomics is failing on diversity. Nature 2016;538:161–4. 27734877 10.1038/538161a PMC5089703

[btaf391-B37] Poplin R , Chang P-C, Alexander D et al A universal SNP and small-indel variant caller using deep neural networks. Nat Biotechnol 2018;36:983–7. 30247488 10.1038/nbt.4235

[btaf391-B38] Samur MK , Aktas Samur A, Fulciniti M et al Genome-wide somatic alterations in multiple myeloma reveal a superior outcome group. J Clin Oncol 2020;38:3107–18. 32687451 10.1200/JCO.20.00461 PMC7499613

[btaf391-B39] Siegel RL , Giaquinto AN, Jemal A. Cancer statistics, 2024. CA: Cancer J Clin 2024;74:12–49. 38230766 10.3322/caac.21820

[btaf391-B40] Sirugo G , Williams SM, Tishkoff SA. The missing diversity in human genetic studies. Cell 2019;177:1080. 31051100 10.1016/j.cell.2019.04.032

[btaf391-B41] Skerget S , Penaherrera D, Chari A et al; MMRF CoMMpass Network. Comprehensive molecular profiling of multiple myeloma identifies refined copy number and expression subtypes. Nat Genet 2024;56:1878–89. 39160255 10.1038/s41588-024-01853-0 PMC11387199

[btaf391-B42] Sudmant PH , Rausch T, Gardner EJ et al; 1000 Genomes Project Consortium. An integrated map of structural variation in 2,504 human genomes. Nature. 2015;526:75–81. 26432246 10.1038/nature15394 PMC4617611

[btaf391-B43] Tang H , Peng J, Wang P et al Estimation of individual admixture: analytical and study design considerations. Genet Epidemiol 2005;28:289–301. 15712363 10.1002/gepi.20064

[btaf391-B44] Weiss BM , Abadie J, Verma P et al A monoclonal gammopathy precedes multiple myeloma in most patients. Blood 2009;113:5418–22. 19234139 10.1182/blood-2008-12-195008 PMC2689043

[btaf391-B45] Yusuf S , Wittes J. Interpreting geographic variations in results of randomized, controlled trials. N Engl J Med 2016;375:2263–71. 27959693 10.1056/NEJMra1510065

