# Supplementary material for: AncestryGeni: a novel genetic ancestry classification pipeline for small and noisy sequence data
Source: Bioinformatics. 2025 Jul 8;41(7):btaf391. doi: 10.1093/bioinformatics/btaf391 (PMC12289551; doi:10.1093/bioinformatics/btaf391)
Supplement: btaf391_Supplementary_Data [file btaf391_supplementary_data.docx]

# Supplementary Note

## Running AncestryGeni

## Overview

AncestryGeni is a modular ancestry inference pipeline that integrates unsupervised ancestry estimation with supervised geographic classification. The pipeline supports human genome builds GRCh37 and GRCh38 and is compatible with a variety of sequencing data types, including whole-genome sequencing (WGS), whole-exome sequencing (WES), and RNA-Seq.

The pipeline operates in two stages:

- First Stage: Identify global gene pools using *unsupervised* ADMIXTURE on reference individuals, followed by creating artificial individuals corresponding to those ancestries, and, finally, *supervised* ADMIXTURE to estimate ancestry proportions of test samples with respect to the artificial individuals, i.e., gene pools.
- Second Stage: Applies a supervised machine learning model to identify continental affiliations based on inferred ancestry proportions.

## 1. First Stage: ADMIXTURE

### 1.1 Rationale

The training process involves two main parts:
1. *Unsupervised* ADMIXTURE is applied to a subset of reference individuals from the 1000 Genomes Project to identify global gene pools (ancestry components). Next, artificial individuals corresponding to those ancestries are created based on the program’s P file that contains the average allele frequencies of individuals represented in that gene pool per SNP.
2. *Supervised* ADMIXTURE estimates the ancestry proportions of each test sample based on these predefined components.

These proportions serve as input features for the second stage, where a supervised machine-learning model classifies continental group affiliations.

### 1.2 Files Needed

Reference Population Data – *ReferencePops.zip*

Includes genetic data in VCF and PLINK formats, along with population labels, metadata, and geographic coordinates.

Archive contains:

• VCF files (Variant Call Format)

• PLINK files (.bed, .bim, .fam)

• Population metadata files

• Geographic coordinates

### 1.3 VCF to PLINK Conversion

To convert a VCF file to PLINK format, run:

plink --vcf input.vcf --make-bed --out output_prefix

This command generates .bed, .bim, and .fam files from the input VCF.

### 1.4 Configuration Files

*Parameters.txt* – Specifies key variables:

ADMIXTURE_DIR=/path/to/admixture
INPUT_DIR=/path/to/vcf/files
OUTPUT_DIR=/path/to/output
OUTPUT_DIR_1KG=/path/to/1kg/output
OUTPUT_DIR_FINAL=/path/to/final/output
OUTPUT_ADMIXTURE_DIR=/path/to/admixture/output
OUTPUT_FILE_TABLE=ancestry_counts.txt
OUTPUT_FILE=snp_counts.txt
DB_NAME=my_dataset
NUM_OF_LINES=1

*config.json* – ML model configuration file:

{
 "INPUT_TRAINING_FOLDER": "./training_data",
 "INPUT_TESTING_FOLDER": "./testing_data",
 "INPUT_TRAINING_FILE": "training.txt",
 "INPUT_TESTING_FILE": ["test.txt"],
 "SPLIT_DATA": 1,
 "MMRF_DATA": 0,
 "N_SPLITS": 10,
 "COLS": {
 "TARGET": ["Code"],
 "ANNOT": ["Dataset", "Sample", "SampleCode"]
 }
}

### 1.5 Commands

To set up and run the pipeline:

git clone https://github.com/eelhaik/AncestryGeni.git

cd AncestryGeni

cd pipeline_setup

bash Run_AncestryGeni.txt

## 2. Second Stage: Classification (ML)

### 2.1 Basic Classification – ClassifyGeoGroup1.py

cd ../ClassifyGeoGroup_ML_model

python ClassifyGeoGroup1.py

Performs initial ancestry classification using any number of continental groups. This is the first stage of the ancestry classification pipeline, designed to provide a quick and efficient initial assessment of continental ancestry. The current script focuses on broad ancestry categories, assigning individuals to major continental groups such as European, African, Asian, and “others.” It generates a confusion matrix to evaluate performance and provides straightforward ancestry assignments without probability scores. This stage is particularly useful when you need a rapid overview of ancestry composition or when working with large datasets where computational efficiency is important. The output is simpler and more direct, making it ideal for initial screening or when 2-ways continental ancestry breakdowns and probabilities are not required.

### 2.2 Detailed Classification – ClassifyGeoGroup2.py

python ClassifyGeoGroup2.py

This second stage of the pipeline performs 2-ways continental ancestry classification with probability scores and visualizations. The script provides probability scores for each ancestry classification, identifying not just the most likely ancestry but also alternative possibilities. It is particularly valuable for analyzing admixed populations or individuals with complex ancestry backgrounds, as it can detect and flag borderline cases where ancestry assignment is unclear. The script generates extensive visualizations, including probability heatmaps and detailed reports, making it the preferred choice for in-depth ancestry analysis, research purposes, or when dealing with populations with significant genetic admixture.

### 2.3 Visualization Tools – lda_performance_viz.py

Provides ROC and PR curve visualizations:

cd ClassifyGeoGroup_ML_model/tuning_and_vis

python lda_performance_viz.py --input ../results/classification.csv --output ./visualizations/

### 2.4 Usage Flow

Recommended order of operations:

1. Run ClassifyGeoGroup1.py for initial ancestry classification

2. Run ClassifyGeoGroup2.py for deeper analysis

3. Visualize more using lda_performance_viz.py

## 3. Toy Dataset Usage

### 3.1 Files Needed

*mixed_samples.xlsx* – Excel file with ancestry components, labels, and metadata

Example data:

| Sample_Name | Original_HGDP_IDs | True_Ancestry | Classified_Ancestry | Top1_classification | Top1_Probability | Top2_classification | Top2_Probability |
| --- | --- | --- | --- | --- | --- | --- | --- |
| HGDP00336 | HGDP00336 | Europe-Europe | Europe-Europe | Europe-Europe | 0,554318 | Africa-Europe | 0,445644 |
| HGDP00503_HGDP00444 | HGDP00503_HGDP00444 | America-East Asia | America-East Asia | America-East Asia | 1 | America-Europe | 7,71E-17 |
| HGDP00613_HGDP00869 | HGDP00613_HGDP00869 | Africa-East Asia | Africa-East Asia | Africa-East Asia | 0,999937 | East Asia-Europe | 6,33E-05 |
| HGDP00662_HGDP00448 | HGDP00662_HGDP00448 | East Asia-Europe | East Asia-Europe | East Asia-Europe | 0,931188 | Africa-East Asia | 0,068812 |

• Important Notes:

1. All ancestry proportions must be decimal numbers between 0 and 1.
2. Values use commas as decimal separators (European format).
3. The sum of ancestry components must equal 1.0.
4. Sample_Name and Final_HGDP are identifiers.
5. Ancestry_Label indicates the known ancestry group.

### 3.2 Commands

cd ClassifyGeoGroup_ML_model

python ClassifyGeoGroup2.py --input Toy_dataset/mixed_samples.xlsx

## 4. Output Files

### 4.1 First Stage Outputs

• *.Q: Ancestry proportions
• *.P: Allele frequencies
• *.log: Convergence and runtime info

Note: The .Q file is used as input for the ML part.

Example: 0.304622 0.538934 0.002897 ... 0.00001

### 4.2 Second Stage Outputs

• *_with_classification.csv* – Classifiedancestry
• *output_basic.txt* / output_detailed.txt – Metrics
*• best_model.pkl* – Saved model
• Visualizations – Confusion matrix, heatmaps

## 6. Performance Metrics for the testing set

• Accuracy
• F1-score
• Precision/Recall
• Cohen's kappa
• MCC

## 7. System Requirements

• Python 3.x (with numpy, pandas, scikit-learn, joblib, commentjson)
• ADMIXTURE software
• 1–2 GB RAM for classification
• 4–8 CPU threads recommended
• 8 GB RAM for ADMIXTURE
